# Multimodal Retinal Imaging for the Detection of Early Alzheimer’s Disease: Combining Biochemical, Structural, and Vascular Biomarkers

**DOI:** 10.3390/bioengineering13091041

**Published:** 2026-09-08

**Authors:** Michiel Ghesquiere, Eirini Christinaki, Sophie Lemmens, Jan Van Eijgen, Lennert Beeckmans, Achilleas Ghinis, Thomas Jacobs, Karel Van Keer, Zahi Wehbi, Lies De Groef, Yasmin Dahdouh-Guebas, Wouter Charle, Thomas Vande Casteele, Mathieu Vandenbulcke, Greet Vanderlinden, Koen Van Laere, Jolien Schaeverbeke, Rik Vandenberghe, Jenny Ceccarini, Maarten De Vos, Ingeborg Stalmans

**Affiliations:** 1 Research Group Ophthalmology, Department of Neurosciences, KU Leuven, ON5 Herestraat 49, 3000 Leuven, Belgium; michiel.ghesquiere@gmail.com (M.G.); eirini.christinaki@kuleuven.be (E.C.); sophie.1.lemmens@uzleuven.be (S.L.); jan.1.vaneijgen@uzleuven.be (J.V.E.); lennert.beeckmans@kuleuven.be (L.B.); achilleas.ghinis@kuleuven.be (A.G.); thomas@drjacobs.be (T.J.); karel@vankeer.org (K.V.K.); zahitwehbi@gmail.com (Z.W.); 2 Dynamical Systems, Signal Processing and Data Analytics (STADIUS), Department of Electrical Engineering (ESAT), KU Leuven, Kasteelpark Arenberg 10, 3001 Leuven, Belgium; maarten.devos@kuleuven.be; 3 Department of Ophthalmology, University Hospitals UZ Leuven, 3000 Leuven, Belgium; 4 Public Health and Primary Care, Kulak Kortrijk Campus, KU Leuven, Etienne Sabbelaan 53, 8500 Kortrijk, Belgium; 5 Department of Biology, Animal Physiology and Neurobiology, KU Leuven, Naamsestraat 59, 3000 Leuven, Belgium; lies.degroef@kuleuven.be (L.D.G.); yasmin.dahdouh-guebas@kuleuven.be (Y.D.-G.); 6 Imec, Kapeldreef 75, 3001 Leuven, Belgium; wouter.charle@imec.be; 7 Department of Neurosciences—Neuropsychiatry, Leuven Brain Institute, KU Leuven, UZ Herestraat 49, 3000 Leuven, Belgium; thomas.vandecasteele@upckuleuven.be (T.V.C.); mathieu.vandenbulcke@uzleuven.be (M.V.); 8 Geriatric Psychiatry, University Psychiatric Center KU Leuven, 3000 Leuven, Belgium; 9 Nuclear Medicine and Molecular Imaging, Department of Imaging and Pathology, KU Leuven and University Hospitals Leuven, UZ Herestraat 49, 3000 Leuven, Belgium; greet.vanderlinden@uzleuven.be (G.V.); koen.vanlaere@uzleuven.be (K.V.L.); 10 Laboratory of Neuropathology, Department of Imaging and Pathology, Leuven Brain Institute (LBI), KU Leuven, 3000 Leuven, Belgium; jolien.schaeverbeke@kuleuven.be; 11 Laboratory for Cognitive Neurology, Department of Neurosciences, Leuven Brain Institute (LBI), KU Leuven, 3000 Leuven, Belgium; rik.vandenberghe@uzleuven.be; 12 Alzheimer Research Center KU Leuven, Leuven Brain Institute, Herestraat 49, 3000 Leuven, Belgium; 13 Department of Neurosciences, Leuven Brain Institute, KU Leuven, Biomedical Sciences Group, ON5 Herestraat 49, 3000 Leuven, Belgium; jenny.ceccarini@kuleuven.be; 14 Department of Development and Regeneration, Woman and Child, KU Leuven, Herestraat 49, 3000 Leuven, Belgium

**Keywords:** Alzheimer’s disease, retinal imaging, hyperspectral imaging, optical coherence tomography, vascular biomarkers, machine learning

## Abstract

Alzheimer’s disease (AD) pathology is increasingly recognized to manifest in the retina, offering a non-invasive window for early biomarker discovery. This proof-of-concept study investigated whether multimodal retinal imaging—hyperspectral imaging (HSI), optical coherence tomography (OCT), and color fundus photography (CFP)—can differentiate individuals with and without cerebral amyloid-beta (Aβ) pathology in 40 participants with PET-confirmed Aβ status (17 Aβ+, cognitively normal or with mild cognitive impairment; 23 Aβ− cognitively normal controls). HSI-derived gray-level co-occurrence matrix (GLCM) texture, OCT-derived ganglion cell–inner plexiform layer (GC-IPL) thickness, and CFP-derived vascular biomarkers (VBMs) were extracted, and logistic regression with leave-one-out cross-validation assessed classification performance per modality, alone and combined; the cohort was supplemented with AD dementia patients for an exploratory cross-sectional comparison across disease-stage groups. HSI showed nominally lower GLCM correlation at 466 nm in Aβ+ participants, most pronounced in the inferior macula (AUC = 0.72). GC-IPL thickness showed a similar inferior-predominant regional pattern. Combining HSI and GC-IPL features yielded the best performance (AUC = 0.84; sensitivity = 0.82; specificity = 0.78), although this improvement over the unimodal models did not reach statistical significance, whereas vascular biomarkers contributed minimally. In an exploratory cross-sectional comparison across AD stage groups drawn from two cohorts, HSI features showed a non-monotonic pattern, decreasing in early Aβ+ stages and rising again in dementia. These findings provide preliminary evidence of complementary information between HSI and OCT for detecting retinal biomarkers of early-stage AD, supporting multimodal retinal imaging as a scalable screening approach warranting validation in larger, longitudinal cohorts.

## 1. Introduction

Dementia is a major public health concern, with more than 150 million people expected to be affected by 2050 due to demographic aging [1]. Alzheimer’s disease (AD), the leading cause of dementia, progresses along a biological continuum that begins decades before clinical symptoms emerge, marked by the silent accumulation of amyloid-β (Aβ) and tau [2]. As the disease progresses, these protein aggregates trigger synaptic dysfunction and neuronal loss, alongside cerebral amyloid angiopathy, neuroinflammation, and gliosis, reflecting the multifactorial nature of the disease [3]. Current global estimates suggest that approximately 315 million individuals are in the preclinical phase (Aβ-positive but cognitively normal, CN), 69 million in the prodromal stage (mild cognitive impairment, MCI due to AD), and over 32 million live with AD dementia (ADD) [4]. Given this large at-risk population, there is an urgent need for effective early-detection strategies. The recent arrival of monoclonal antibodies as a promising therapeutic approach, showing moderate effect in decelerating cognitive decline, further underscores the importance of timely identification and intervention [5]. Moreover, early biomarker-based identification not only facilitates timely lifestyle interventions but also enables clinical trial recruitment, which is crucial for advancing the development of disease-modifying treatments [6,7]. Furthermore, understanding biomarker trajectories across the disease continuum is crucial for monitoring disease progression [8]. However, current gold-standard biomarkers, such as cerebrospinal fluid analysis (CSF) assays and positron emission tomography (PET), are invasive, expensive, and unsuitable for population-level screening [9]. This has driven efforts towards non-invasive, scalable, and cost-effective alternatives [10], including promising blood-based biomarkers (BBMs) such as plasma phosphorylated tau (p-tau), which demonstrate high diagnostic accuracy [11].

**Figure 1:**
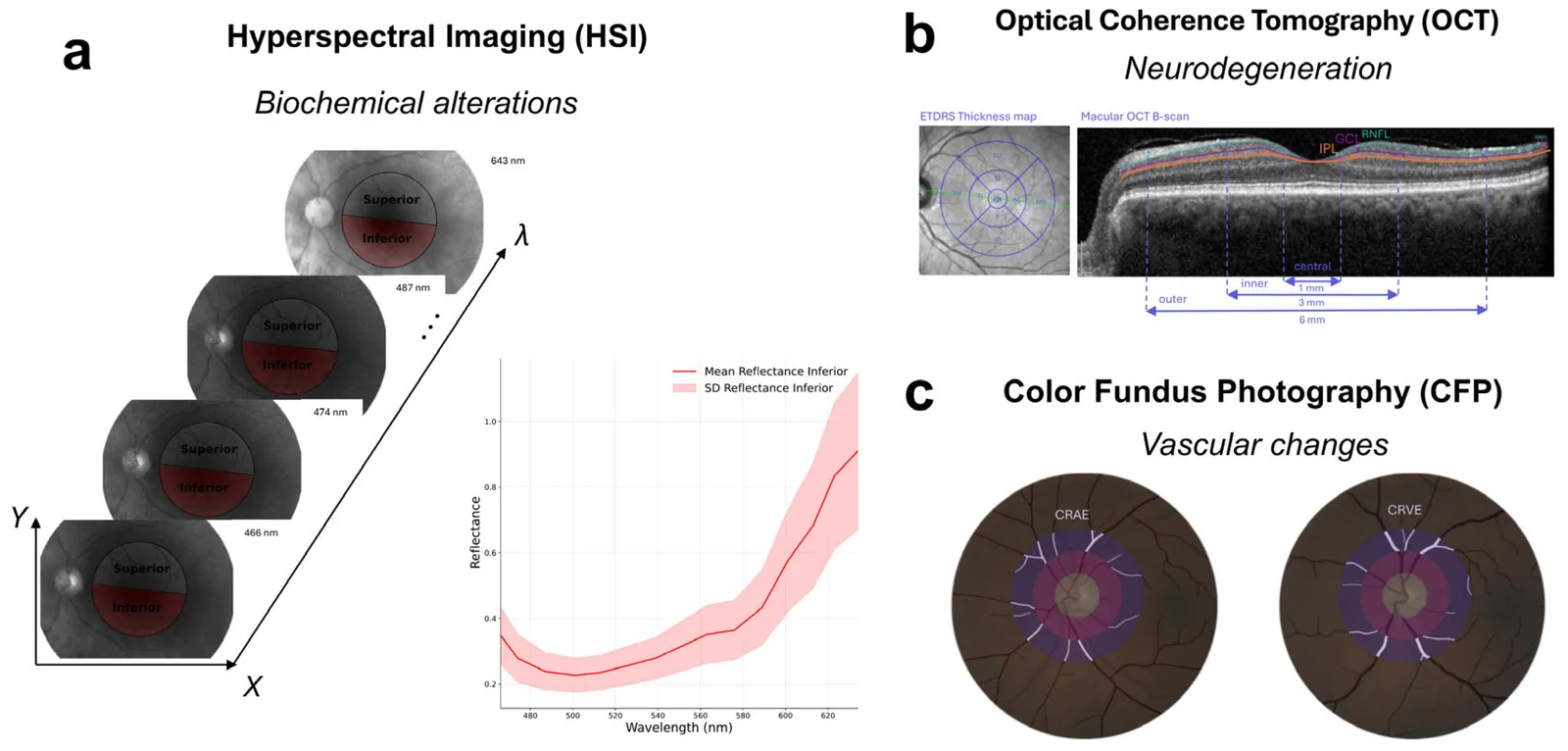
Multimodal retinal images from a cognitively normal, Aβ− subject. (**a**) HSI captures both spatial and spectral information, enabling biochemical analysis of retinal structures. The mean and SD reflectance values of pixels in the inferior hemisphere are shown. (**b**) OCT provides structural measurements, including RNFL, GC and IPL thickness, across the regions of interest defined by the ETDRS grid, indicative of neurodegeneration. (**c**) CFP enables VBM extraction via DL-based artery and vein segmentation. As shown, arteriolar (**left**) and venular (**right**) diameters can be quantified using CRAE and CRVE, respectively. Aβ = amyloid-beta; HSI = hyperspectral imaging; SD = standard deviation; OCT = optical coherence tomography; retinal nerve fiber layer; GC = ganglion cell layer; IPL = inner plexiform layer; ETDRS = Early Treatment Diabetic Retinopathy Study; CFP = color fundus photography; VBM = vascular biomarker; DL = deep learning; CRAE = central retinal arteriolar equivalent; CRVE = central retinal venular equivalent.

Alternatively, due to its common embryological origin and direct anatomical continuity with the brain, the retina has gained prominence as a non-invasive biomarker site for detecting early AD-related changes [12,13,14]. Among the available retinal imaging modalities, hyperspectral imaging (HSI) offers a unique advantage by capturing both spatial and spectral information (Figure 1a), enabling detection of subtle biochemical alterations that may precede any discernible structural damage [15,16]. Furthermore, both postmortem and in vivo studies have demonstrated that HSI can identify spectral signatures associated with retinal Aβ deposition [15,16,17]. To mitigate the high inter-subject variability of raw spectral reflectance data [18,19], recent studies have incorporated texture features derived from the gray-level co-occurrence matrix (GLCM), which capture spatial relationships between pixel intensities and improve discrimination robustness [19]. Nonetheless, the potential of HSI remains underexplored in AD research. The macula has been suggested as a particularly susceptible site in preclinical AD, with several studies reporting subtle thinning of macular layers in individuals with MCI [20,21,22]. These findings indicate that the macula may exhibit early pathological changes, potentially before widespread neurodegeneration is detectable, which could be more sensitively captured through GLCM-based texture analysis [23]. Yet, no HSI study to date has systematically examined how texture alterations vary across the macula, representing a critical step toward identifying regions of early retinal vulnerability in AD. Moreover, establishing the measurement reliability of such novel features is critical for their validation as clinical biomarkers.

Adding to the biochemical insights from HSI, other complementary retinal imaging biomarkers can capture different aspects of AD pathology, such as structural and vascular changes. Optical coherence tomography (OCT) provides high-resolution cross-sectional imaging of the retina, enabling quantification of structural neurodegeneration through layers such as the retinal nerve fiber layer (RNFL) consisting of ganglion cell axons, the ganglion cell layer (GC) containing their cell bodies, and the inner plexiform layer (IPL) that includes their dendrites and synapses (Figure 1b) [22]. Standard OCT protocols often quantify the latter two as a single unit, the ganglion cell-inner plexiform layer (GC-IPL), which has emerged as a sensitive biomarker of neurodegeneration in MCI and AD [24]. In parallel, vascular biomarkers (VBMs) can shed light on the microvascular dysfunction known to contribute to AD progression [25].

Advances in machine learning (ML) and deep learning (DL) techniques are increasingly used for automated biomarker extraction and AD classification across neuroimaging and ophthalmic imaging modalities [26,27]. Using DL-based segmentation of color fundus photography (CFP), VBMs such as the Central Retinal Arteriolar and Venular Equivalents (CRAE/CRVE) can be automatically extracted for large-scale analysis (Figure 1c) [28,29].

Prior studies have demonstrated the added value of combining imaging modalities. For instance, Sharafi et al. [19] used a support vector machine (SVM) to show that supplementing vascular measures with HSI-based texture features improved detection accuracy from 76% to 85%. Similarly, in our previous work, we demonstrated that a linear discriminant analysis (LDA) model combining HSI with OCT features outperformed HSI alone, enabling more accurate classification of individuals with established ADD [30]. More broadly, recent work in AD neuroimaging has increasingly focused on multimodal fusion, leveraging complementary information across imaging modalities to improve disease characterization and classification [31]. Despite these insights, it remains unknown whether this diagnostic ability and synergy extend to the more subtle pathological changes present in early, Aβ PET-confirmed stages of AD. A comprehensive model integrating biochemical (HSI), structural (OCT), and vascular (CFP-based) biomarkers for early AD detection has yet to be systematically developed and validated.

To address these knowledge gaps, we conducted a proof-of-concept study focused on early AD detection and exploratory cross-sectional retinal biomarker patterns across disease stages. Our primary classification analyses were performed in a well-characterized cohort of CN and MCI participants with PET-confirmed Aβ status. For an exploratory comparison across the AD continuum, this cohort was supplemented with a group of patients with ADD from an independently recruited cohort.

Our aims were threefold: (1) to evaluate HSI-derived texture features for classifying Aβ status and to identify candidate topographic patterns associated with cerebral amyloid status; (2) to explore the cross-sectional pattern of the most salient HSI biomarker across disease-stage groups using the augmented cohort; and (3) to systematically compare the classification performance of optical HSI, structural OCT, and CFP-derived vascular biomarkers, both individually and in combination.

## 2. Materials and Methods

### 2.1. Study Participants and Cohort Definitions

To address the study aims, two distinct participant cohorts were included. The primary CN/MCI cohort was used for unimodal and multimodal classification analyses, while an additional CN/ADD cohort was combined with the primary cohort for exploratory, cross-sectional analysis of biomarker differences across the AD continuum.

The primary CN/MCI cohort consisted of 40 participants recruited for the prospective, longitudinal biomarker study Multimodal Retinal Imaging in the Detection and Follow-up of Alzheimer’s Disease (RetAD) (ClinicalTrials.gov: NCT03466177). We included participants aged 50–85 years with complete multimodal retinal imaging and Aβ PET data. For each participant, a single study eye was selected and used consistently for all imaging modalities and follow-up visits. Exclusion criteria included major neurological disorders other than AD, large-vessel stroke, focal brain lesions on MRI, or ocular diseases other than refractive error or cataract (e.g., glaucoma). Participants underwent a standardized neuropsychological assessment to evaluate global cognition (Mini-Mental State Examination [MMSE]) and specific domains including memory (Auditory Verbal Learning Test [AVLT]), language (Boston Naming Test [BNT]), and verbal fluency (Animal Verbal Fluency [AVF]). Amyloid burden was assessed via static ^18^F-Flutemetamol PET scans acquired 90–110 min post-injection [32]. Standardized uptake value ratios (SUVRs) were converted to Centiloid (CL) values, with participants classified as Aβ positive (Aβ+) if CL values  >  23.5 and Aβ negative (Aβ−) if CL ≤ 23.5 [33]. APOE genotyping was performed on venous blood samples collected at baseline, with sequencing performed at the Genetic Service Facility (GSF, www.vibgeneticservicefacility.be, accessed on 27 August 2026), VIB Department of Molecular Genetics [34]. Study protocols were approved by the Ethics Committee Research UZ/KU Leuven and were conducted in accordance with the EU Directive on Clinical Trials (2001/20/EC) and the Declaration of Helsinki.

For this exploratory cross-sectional analysis, the primary cohort was supplemented with an additional CN/ADD cohort from our previously published study [30], comprising 22 CN controls and 17 patients with clinically or biomarker-confirmed ADD, with no overlap in participants between the cohorts.

### 2.2. Image Acquisition and Feature Extraction

#### 2.2.1. Hyperspectral Retinal Imaging (HSI)

Acquisition and pre-processing

Macula-centered hyperspectral retinal images were acquired using a MQ022HG-IM-SM4X4-VIS2 snapshot camera (Ximea, Münster, Germany) coupled to a Topcon TRC-50DX fundus camera (Topcon Corporation, Tokyo, Japan). The system acquired 16 spectral bands spanning approximately 460–620 nm in a single 0.2 ms exposure over a 50° field of view, with a 10-bit depth and analog gain of 3.2× [35,36]. Acquisition and radiometric pre-processing followed the protocol described by Lemmens et al. [30]. Raw hyperspectral measurements were converted to relative reflectance using dark- and white-reference acquisitions. For each pixel and wavelength, relative reflectance was calculated as

(1) $$R_{i}=\frac{{RS}_{i}-{RD}_{i}}{{RW}_{i}-{RDW}_{i}},$$

where *RS_i_* denotes the raw sample intensity, *RD_i_* the dark reference acquired with the illumination source disabled and the lens covered, *RW_i_* the white reference obtained by imaging a standard white Spectralon target, and *RDW_i_* a dark reference acquired using the same exposure time as the white reference. Sample and corresponding dark-reference images were acquired at 0.2 ms, whereas the white reference and its matched dark reference were acquired at 0.15 ms to avoid saturation [30]. Because fundus-camera flash intensity was adjusted between participants to maximize retinal signal without saturation, absolute reflectance was not estimated; subsequent within-image standardization was used to reduce residual inter-subject differences in illumination.

Spectral correction was then performed using the sensor-specific calibration and preprocessing software supplied by imec for the SNm4x4 VIS snapshot sensor. This proprietary calibration implements the camera-specific correction approach described in [37] to compensate for spectral cross-talk, leakage, harmonics, and non-ideal spectral-filter responses. After correction, 14 spectral bands were retained for analysis, with two channels excluded because of insufficient signal-to-noise characteristics, consistent with the previously validated preprocessing pipeline [30]. For the present study, retinal vessel removal was performed using a deep-learning-based vessel segmentation approach [28], rather than the difference-of-Gaussians procedure used in the earlier Lemmens et al. pipeline. The resulting vessel masks were applied before extraction of HSI reflectance and texture features, as described below.

Participants from both the CN/MCI and CN/ADD cohorts were imaged using the same HSI hardware and nominal acquisition and preprocessing protocol. This was intended to maximize technical comparability between cohorts; however, because the cohorts were recruited and acquired separately, residual cohort- or acquisition-related effects cannot be excluded.

Quality control and regions of interest (ROIs)

Each pre-processed HSI underwent rigorous visual inspection, including evaluation of all 14 spectral bands, a pseudo-RGB reconstruction approximating a color fundus image, and the corresponding vessel segmentation mask generated by a DL model [28]. Images with poor vessel delineation [38], significant motion blur, or illumination artifacts were excluded from further analysis. For analysis of these high-quality images, a 6 mm circular macular grid was applied, corresponding to the diameter of the Early Treatment Diabetic Retinopathy Study (ETDRS) grid [39]. The optic disc (OD) center was automatically detected using a DL model [29]. This grid was centered on the fovea, which was manually annotated by an ophthalmologist on a pseudo-RGB HSI reconstruction and was subsequently checked by a second reviewer. This macular region was then divided into superior and inferior hemispheres using the line connecting the OD center and fovea as the boundary. To ensure consistent ROI sizing despite slight variations in eye position and field of view, the physical distance between the OD center and fovea (~4.5 mm) was mapped to pixel coordinates. These ROIs were then applied uniformly across all 14 spectral channels, after masking out retinal vessels [28].

Feature extraction

From each retinal vessel-masked ROI, two distinct feature sets were extracted for each of the 14 spectral bands:

- Normalized spectral reflectance: To minimize inter-subject variability arising from imaging factors (e.g., illumination differences), relative reflectance values within each ROI were standardized using the mean and standard deviation of the entire image, as described by Lemmens et al. [30]. The mean standardized reflectance value within each ROI was then calculated, yielding a 14-dimensional spectral feature vector.
- GLCM texture features: To quantify retinal tissue organization, five second-order statistical descriptors were derived from the Gray Level Co-occurrence Matrix (GLCM): contrast (CON), dissimilarity (DIS), homogeneity (HOM), energy (ENE), and correlation (COR) [23,40]. Using the “*graycomatrix*” function from the *scikit-image* Python package (version 0.23.2), GLCM features were calculated with a pixel-pair distance of 1 (capturing fine-grained local texture), averaged across four orientations (0°, 45°, 90°, and 135°) to ensure rotational invariance, and normalized to allow comparability across ROIs and imaging conditions.

#### 2.2.2. Optical Coherence Tomography (OCT)

Spectral-domain OCT (SPECTRALIS, Heidelberg Engineering) was performed on a single study eye centered on the macula. The Heidelberg Eye Explorer software automatically extracted RNFL and GC-IPL thickness values across the nine-region ETDRS grid. Subsequently, GC-IPL thickness values were used as features, either from all nine regions combined (for the full macular grid) or from subsets corresponding to the HSI grids (e.g., Inner and Outer Inferior regions for the inferior hemisphere analysis).

#### 2.2.3. Color Fundus Photography (CFP)

Disc-centered 30° field-of-view CFP were acquired using the Visucam PRO NM camera (Carl Zeiss Meditec, Jena, Germany). In case of multiple CFPs captured on the same day, the highest-quality image was manually selected for the analysis. Following the same protocol as in Fhima et al. [41], the arterioles and venules were segmented from the CFPs using a DL model, after which VBMs were extracted from the segmentation masks using the Python Vasculature BioMarker (PVBM, v3.0.1.0) toolbox [29]. The CRAE and CRVE were calculated using the Knudtson method [42]. For one subject with discontinuous vessel segmentation, VBMs were imputed using the dataset mean.

### 2.3. Statistical Analysis

Group Comparisons

Differences in retinal features between Aβ− and Aβ+ participants were evaluated using two-sided Mann–Whitney U tests. As this study represents an exploratory biomarker screen aimed at identifying candidate features for classification, group differences were evaluated using unadjusted *p*-values without correction for multiple comparisons [43]. We recognize that this increases the likelihood of false positive findings (Type I error), and accordingly, all reported *p*-values should be interpreted as hypothesis-generating rather than confirmatory. Demographic variables (age and sex) were compared between groups using appropriate statistical tests following normality evaluation. For continuous variables (age), independent sample *t*-tests were used when data followed a normal distribution, otherwise Mann–Whitney U tests were applied. For multiple group comparisons, the Kruskal–Wallis test was used for continuous variables. For categorical variables, chi-square tests were performed for both pairwise and multiple group comparisons. To assess the overall effect of group differences on structural OCT measures (RNFL and GC-IPL), a multivariate analysis of covariance (MANCOVA) was conducted, with age and sex included as covariates, using Wilks’ Lambda to test for significance (*p* < 0.05). As a sensitivity analysis for multiplicity, the exploratory group-comparison *p*-values were also evaluated with the Benjamini–Hochberg false discovery rate (FDR) procedure [44], applied separately within each of five prespecified feature families (42 GLCM COR wavelength-by-region comparisons; 42 normalized-reflectance comparisons; 24 vascular biomarkers; 9 GC-IPL regions; 9 RNFL regions), with results reported as FDR-adjusted q-values.

### 2.4. Repeatability and Reproducibility

To assess the robustness of the HSI-derived texture features (e.g., GLCM COR at 466 nm) across the defined macular ROIs, repeatability and reproducibility analyses were performed. For this, we utilized data from participants within the RetAD cohort who had the necessary repeated image acquisitions. Reliability was quantified using the Intraclass Correlation Coefficient (ICC) based on a two-way random-effects model for absolute agreement (ICC(2,1), pingouin Python package, v0.6.1). Repeatability, defined as test–retest consistency across longitudinal timepoints (e.g., baseline vs. 6-month follow-up) with identical flash settings, was assessed using 20 unique subject-flash pairs from 14 subjects. Reproducibility, defined as consistency across different flash intensities within a single visit, was assessed using 31 distinct image pairs from 22 subjects. The number of image pairs exceeded the number of individual participants because some individuals contributed multiple eligible data points, for instance at different follow-up visits or with various acquisition settings.

### 2.5. Cross-Sectional Biomarker Differences Across AD Stage Groups

For this exploratory cross-sectional analysis, the combined cohort was organized into three distinct groups: (1) CN controls, comprising all Aβ− CN individuals from the primary cohort and the CN controls from the additional cohort; (2) early Aβ+ individuals, representing the preclinical (CN) or prodromal (MCI) group from the primary cohort; and (3) AD patients with clinical or biomarker-confirmed ADD from the additional cohort [30]. GLCM COR at 466 nm (full macular grid) was compared across these three groups. This is a cross-sectional comparison of non-overlapping groups drawn from two cohorts and is not intended to infer longitudinal biomarker change; cohort and disease stage are therefore partially confounded. Group differences in age and sex were also assessed to evaluate potential confounding effects.

### 2.6. Classification and Model Evaluation

Logistic regression classifiers were trained to predict Aβ status (Aβ− vs. Aβ+) using HSI-derived GLCM COR, GC-IPL thickness measures, and CFP-derived vascular biomarkers (VBMs), both individually (unimodal models) and in combination (multimodal models). Based on the exploratory HSI biomarker screen described above, GLCM COR at 466 nm from the full macular grid was used as the HSI predictor in the multimodal analyses.

Model performance was evaluated using leave-one-subject-out cross-validation (LOOCV). Within each fold, feature scaling by z-score normalization was performed using only the training-set statistics and subsequently applied to the held-out participant. Logistic regression models were fitted using L2 regularization, the liblinear solver, a maximum of 1000 iterations, and balanced class weights.

Discrimination was primarily evaluated using the area under the receiver operating characteristic curve (AUC), with accuracy, sensitivity, specificity, precision, and F1-score reported as complementary performance measures [45,46,47]. Ninety-five percent confidence intervals for AUC were estimated from 1000 subject-level bootstrap resamples of the cross-validated predictions.

Given the modest sample size relative to the number of predictors in several models, model complexity was additionally summarized using events per predictor variable (EPV), calculated as the number of Aβ+ participants (n = 17) divided by the number of fitted predictors [48,49,50]. Statistical support for the observed discrimination was evaluated using 1000 label permutations, with the complete LOOCV procedure repeated after each permutation [51,52]. Empirical permutation *p*-values were calculated as p=(b+1)/(B+1), where *b* is the number of permuted AUCs greater than or equal to the observed AUC and *B* = 1000.

Because the HSI+GC-IPL model yielded the highest observed AUC, its performance was compared with that of the alternative models using paired subject-level bootstrap analysis. For each of 1000 bootstrap samples, AUCs were recalculated for both models using the same resampled participants, and the resulting distribution of paired AUC differences (ΔAUC) was used to derive 95% confidence intervals and two-sided *p*-values.

Additional sensitivity analyses evaluated the effects of data-dependent HSI wavelength selection [53], alternative representations of the nine regional GC-IPL measurements, and adjustment for age, sex, and APOE ε4 carrier status. These analyses are described in the Supplementary Methods.

## 3. Results

### 3.1. Participant Characteristics

The primary CN/MCI cohort consisted of 40 adults with PET-confirmed Aβ status. Seventeen participants were Aβ+, including 10 CN and 7 MCI, representing preclinical and prodromal AD stages [2]. The remaining 23 participants were Aβ− CN controls. There were no significant differences in age or sex (Table 1). Cognitive performance differed primarily in the Aβ+ MCI subgroup, which showed lower MMSE scores and worse performance on memory and language measures (auditory verbal learning test, Boston Naming Test, and animal verbal fluency) compared with CN participants. APOE genotype distributions showed a trend toward differences across subgroups. Additional subject-level demographics and cognitive test scores are provided in Supplementary File S2. Demographic characteristics for the additional ADD cohort have been described previously [30].

### 3.2. Hyperspectral Imaging Texture by Macular Region and Amyloid Status

**Figure 2:**
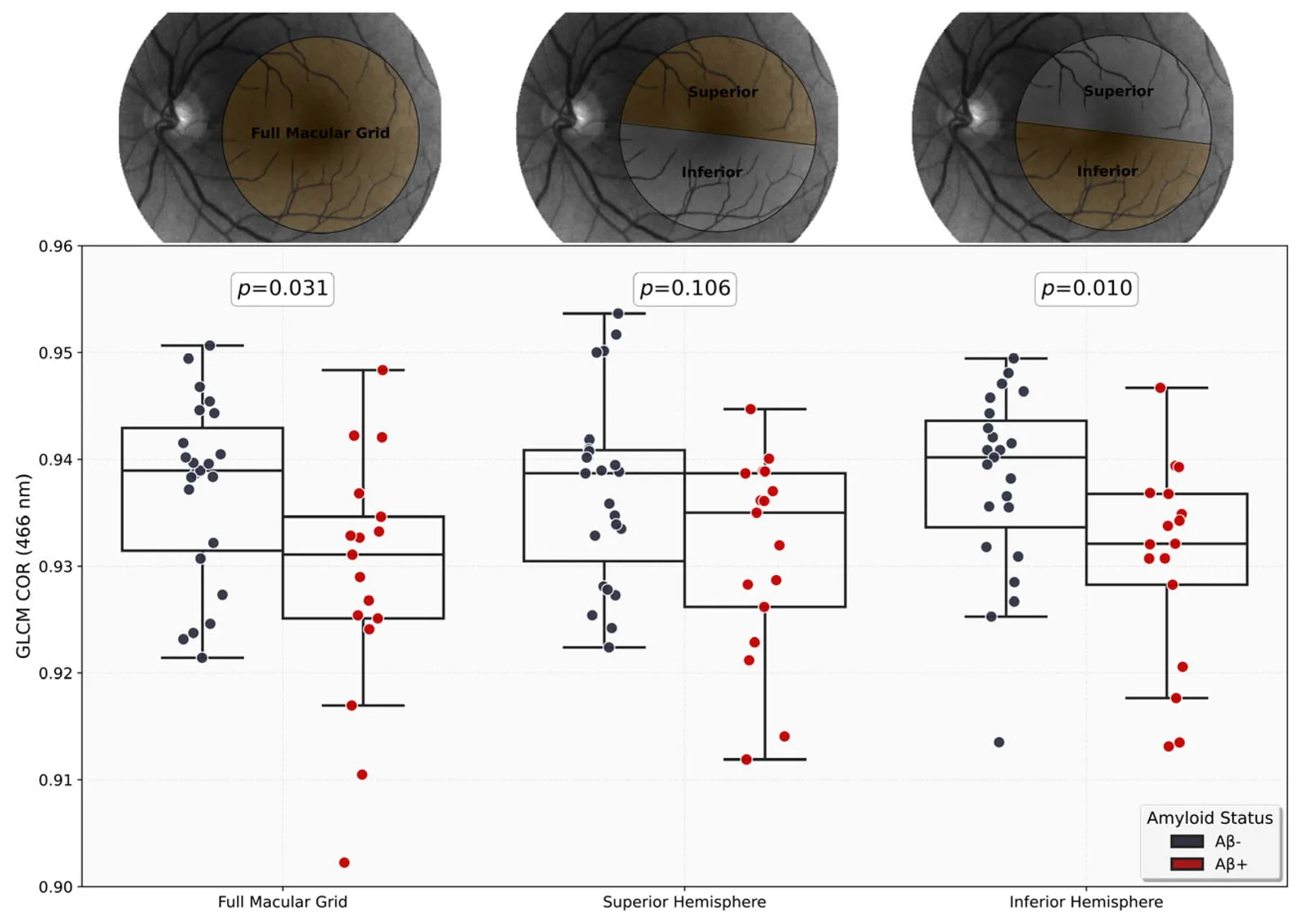
Regional HSI texture analysis by macular region and amyloid status for the primary CN/MCI cohort. HSI texture analysis was performed on the full macular grid, superior hemisphere, and inferior hemisphere. Box plots show the distribution of GLCM COR at 466 nm for Aβ− (blue) and Aβ+ (red) participants. *p* values are computed using the Mann–Whitney U test. Higher GLCM COR values suggest smoother, local texture; lower values indicate more heterogeneous local texture. HSI = Hyperspectral Imaging; CN = cognitively normal; MCI = mild cognitive impairment; GLCM = gray-level co-occurrence matrix; COR = correlation; Aβ+ = amyloid-beta positive; Aβ− = amyloid-beta negative.

Normalized spectral reflectance showed substantial overlap between Aβ− and Aβ+ participants across wavelengths and macular regions (Supplementary Figure S1). In contrast, the exploratory GLCM analysis identified several nominal group differences, with GLCM correlation (COR) at 466 nm showing the strongest candidate signal. Aβ+ participants had lower GLCM COR at 466 nm in the inferior hemisphere (*p* = 0.010) and full macular grid (*p* = 0.031), whereas the difference in the superior hemisphere was not significant (*p* = 0.106) (Figure 2). However, none of the 42 GLCM COR wavelength-by-region comparisons remained significant after Benjamini–Hochberg FDR correction (smallest q = 0.140; Supplementary Table S8).

Using GLCM COR at 466 nm as a single HSI predictor, the inferior hemisphere yielded the highest fixed-feature AUC (0.72; 95% CI 0.54–0.87), followed by the full macular grid (AUC = 0.69; 95% CI 0.50–0.85) and superior hemisphere (AUC = 0.62; 95% CI 0.43–0.79) (Table 2).

Reliability differed by region. Repeatability across longitudinal visits using matched flash settings ranged from ICC 0.48 to 0.60 and was highest for the full macular grid (ICC = 0.60). Residual differences across flash settings may reflect changes in image brightness, local contrast, and pixel-intensity distributions, which are particularly relevant for GLCM COR because it is derived from spatial relationships between neighboring pixel intensities. Reproducibility across different flash intensities within the same visit ranged from ICC 0.64 to 0.88 and was highest for the full macular grid and inferior hemisphere (both ICC = 0.88).

Because the 466 nm wavelength had been identified using the complete cohort, we additionally repeated wavelength selection within each outer LOOCV training fold. For the full macular grid, the resulting HSI-only AUC decreased from 0.69 to 0.52, indicating selection-related optimism in the fixed-feature estimate. Nevertheless, 466 nm remained the most frequently selected wavelength, occurring in 30/40 folds (75%) for the full macular grid and 39/40 folds (97.5%) for the inferior hemisphere (Supplementary Figure S4; Supplementary Table S5). For the inferior hemisphere, fold-internal wavelength selection resulted in a smaller reduction in AUC, from 0.72 to 0.68.

### 3.3. Exploratory Cross-Sectional HSI Pattern Across AD Stage Groups

**Figure 3:**
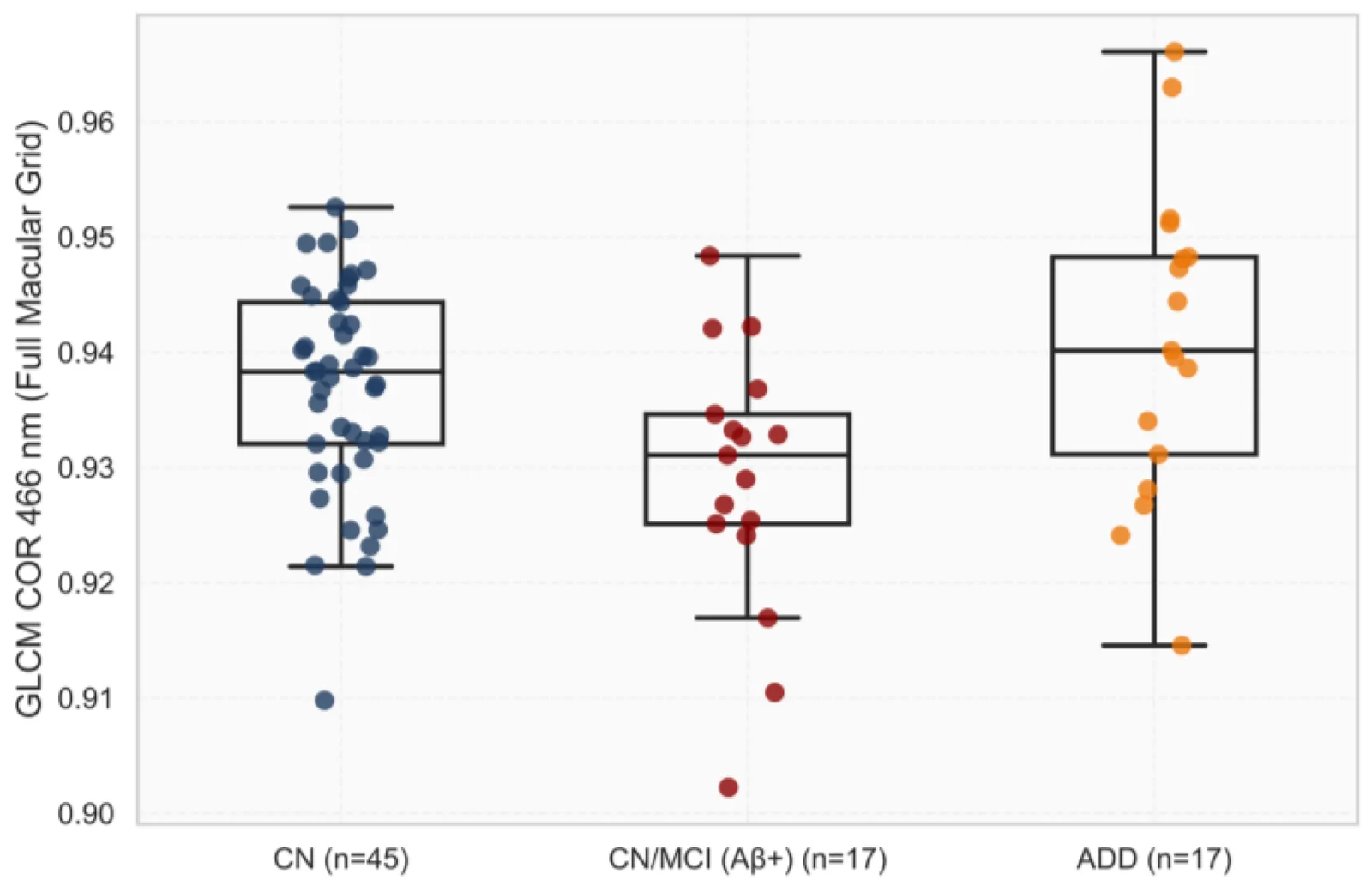
Stage-dependent changes in HSI GLCM COR across the AD continuum. Box plots show the distribution of HSI GLCM COR (full macular grid, 466 nm) across three distinct groups: CN, CN/MCI with Aβ+, and ADD. *p* values are computed using the Mann–Whitney U test. HSI = hyperspectral imaging; GLCM = gray-level co-occurrence matrix; COR = correlation; AD = Alzheimer’s disease; CN = cognitively normal; Aβ = amyloid-beta; MCI = mild cognitive impairment; ADD = Alzheimer’s disease dementia.

In the combined cross-sectional cohort, GLCM COR at 466 nm in the full macular grid was compared across CN controls (n = 45), early Aβ+ participants (CN/MCI; n = 17), and patients with ADD (n = 17). Early Aβ+ participants showed lower GLCM COR than CN controls (*p* = 0.015), whereas the ADD group showed higher values than the early Aβ+ group and did not differ significantly from CN controls (*p* = 0.218) (Figure 3).

To examine this pattern in greater detail, the Aβ+ group was separated into CN,Aβ+ and MCI,Aβ+ participants. Across the four resulting groups, CN,Aβ− (n = 23), CN,Aβ+ (n = 10), MCI,Aβ+ (n = 7), and ADD (n = 17), GLCM COR differed overall (Kruskal–Wallis H = 8.79, *p* = 0.032; Supplementary Figure S7). Relative to CN,Aβ− participants, GLCM COR was not significantly different in CN,Aβ+ participants (*p* = 0.378), was lower in MCI,Aβ+ participants (*p* = 0.029), and was not significantly different in the ADD group (*p* = 0.126).

Because the disease-stage groups originated partly from different cohorts, we also compared CN participants from the two cohorts. GLCM COR at 466 nm did not differ significantly between Aβ− CN participants and CN participants from the Lemmens et al. [30] cohort (*p* = 0.503).

### 3.4. Optical Coherence Tomography-Based Thickness Analysis

Separate MANCOVA analyses were performed for GC-IPL and RNFL thickness, treating measurements from the nine ETDRS regions as multivariate outcomes (Table 3). The overall GC-IPL thickness profile was associated with Aβ status (*p* = 0.009), with sex also contributing significantly (*p* = 0.041). In contrast, the RNFL thickness profile was not associated with Aβ status (*p* = 0.694), although age was significantly associated with RNFL thickness (*p* = 0.024).

At the individual-region level, none of the nine GC-IPL comparisons reached nominal statistical significance (Supplementary Figure S2). Regional RNFL distributions similarly overlapped between Aβ− and Aβ+ participants; descriptive statistics and corresponding FDR-adjusted q-values are provided in Supplementary Figure S5 and Supplementary Table S9.

For classification, the model using all nine GC-IPL regions achieved an AUC of 0.73 (95% CI 0.56–0.88). The inferior regional model (inner and outer inferior sectors) achieved an AUC of 0.71, whereas the corresponding superior model yielded an AUC of 0.45 (Table 4). Sensitivity analyses of the nine-region GC-IPL representation produced similar discrimination with elastic-net regularization (AUC = 0.73) and lower discrimination with fold-internal PCA (AUC = 0.61) (Supplementary Table S6).

### 3.5. Color Fundus Photography-Based Vascular Biomarker Analysis

**Figure 4:**
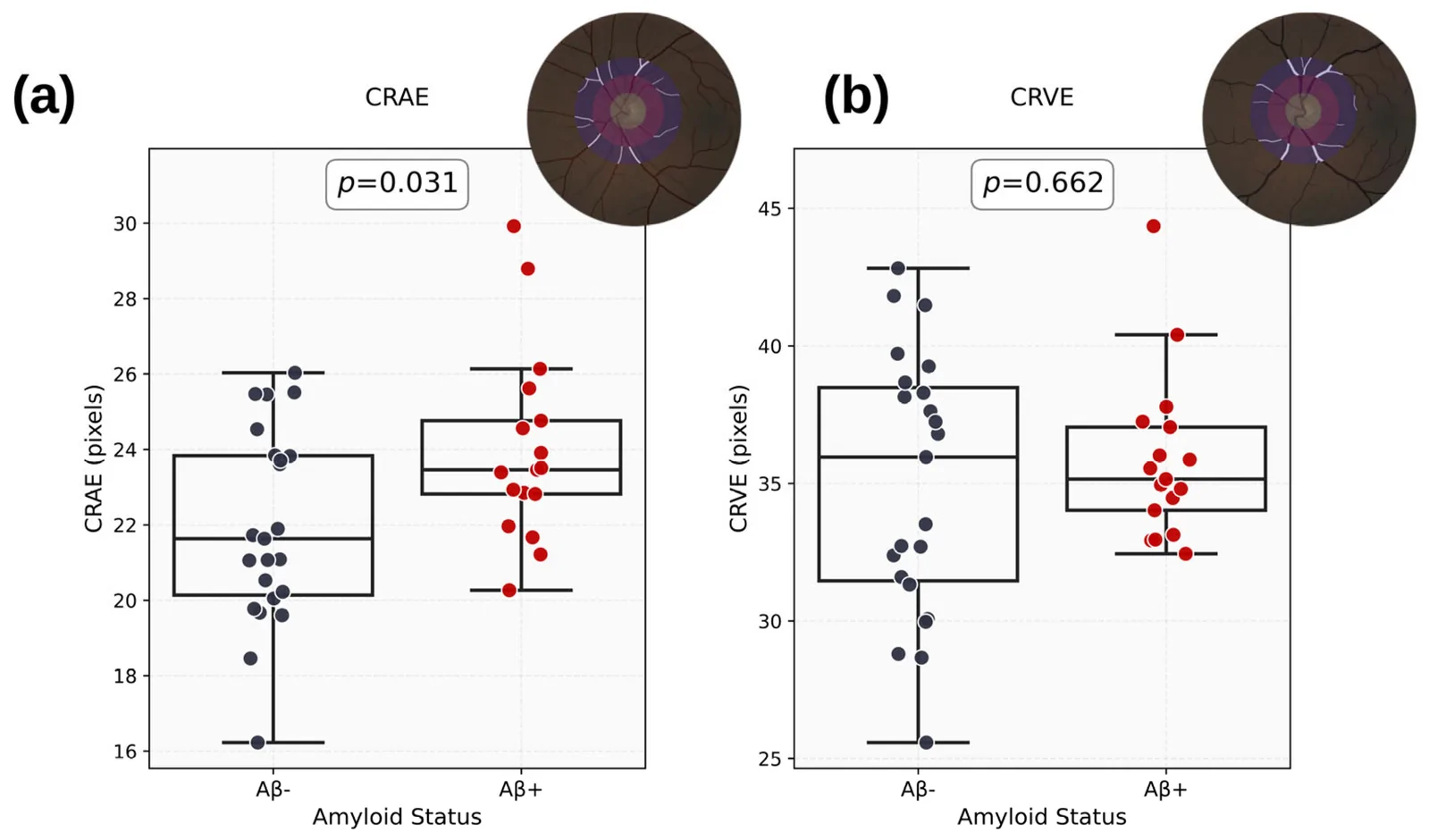
Vascular biomarker distribution by amyloid status. Box plots show the distribution of CRAE (**a**) and CRVE (**b**) for Aβ− (blue) and Aβ+ (red) participants. *p* values are computed using the Mann–Whitney U test. Aβ = amyloid-beta; CRAE = Central Retinal Arteriolar Equivalent; CRVE = Central Retinal Venular Equivalent.

Among the 24 CFP-derived vascular biomarkers, CRAE (*p* = 0.031) and venous endpoint count (*p* = 0.043) showed nominal differences between Aβ− and Aβ+ participants (Figure 4; Supplementary Table S2). Neither remained significant after Benjamini–Hochberg FDR correction (smallest q = 0.519; Supplementary Table S8). The remaining vascular parameters, including tortuosity and fractal-dimension measures, showed no nominal group differences.

For classification, CRAE alone yielded an AUC of 0.66, whereas CRVE yielded an AUC of 0.48. Combining CRAE and CRVE resulted in an AUC of 0.68 (95% CI 0.50–0.84), with sensitivity and specificity of 0.65 (Table 5). The corresponding permutation *p*-value for the combined VBM model was 0.040 (Supplementary Table S4).

### 3.6. Unimodal and Multimodal Retinal Imaging Classification Performance

**Figure 5:**
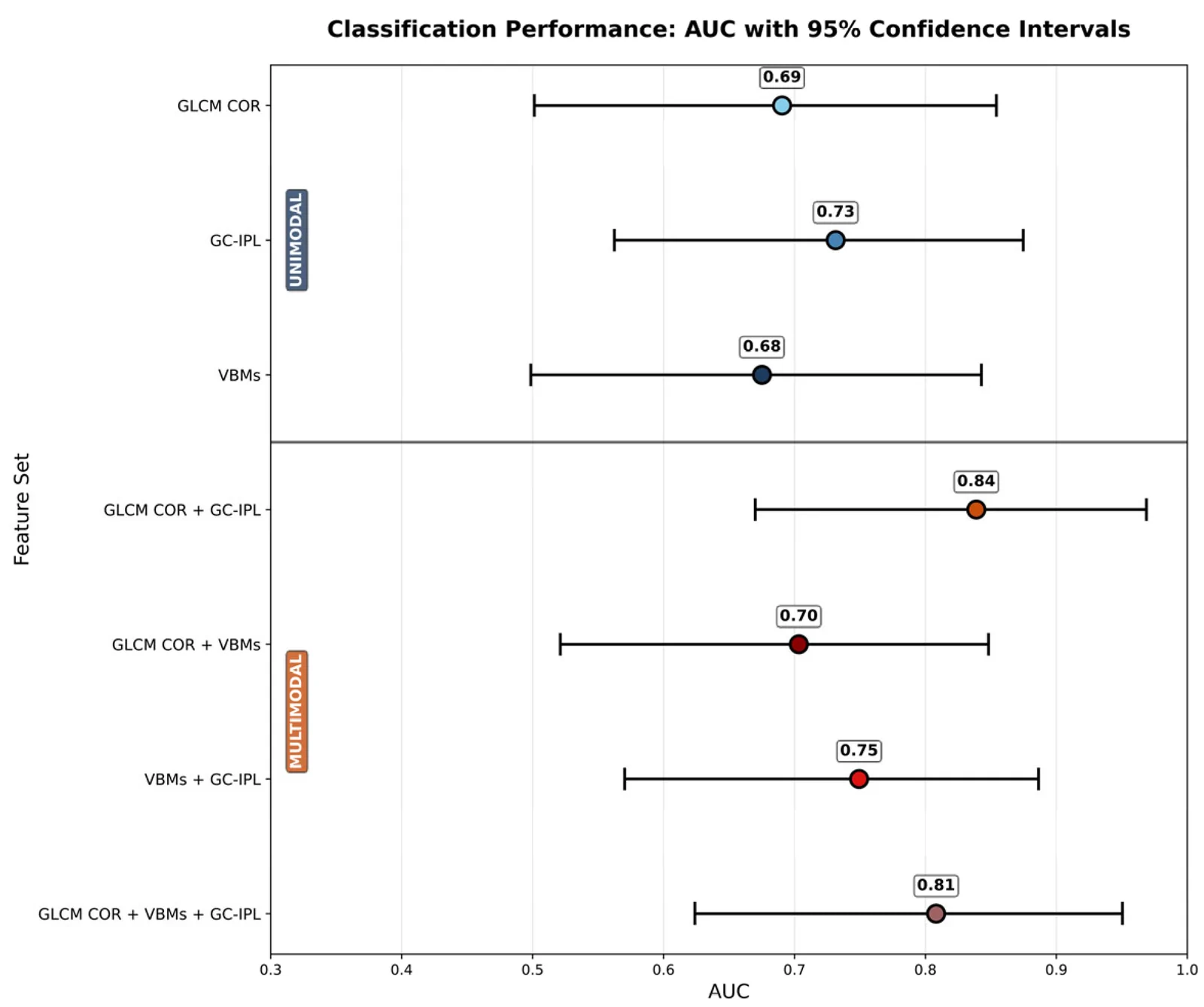
Classification performance (AUC) for unimodal and multimodal feature sets. Bars show AUC values with 95% bootstrap confidence intervals. AUC = area under the curve; HSI = hyperspectral imaging; CRAE = central retinal arteriolar equivalent; CRVE = central retinal venular equivalent; GC-IPL = ganglion cell–inner plexiform layer.

Across the seven full-macular-grid unimodal and multimodal models, the numerically highest cross-validated performance was obtained by combining HSI-derived GLCM COR at 466 nm with the nine regional GC-IPL measurements (AUC = 0.84; 95% CI 0.67–0.97; sensitivity = 0.82; specificity = 0.78) (Figure 5; Supplementary Tables S3 and S4). The corresponding unimodal AUCs were 0.69 for HSI, 0.73 for GC-IPL, and 0.68 for VBMs. Adding CRAE and CRVE to HSI+GC-IPL yielded an AUC of 0.81.

Model complexity varied substantially across feature sets. EPV ranged from 17.00 for the single-feature HSI model to 1.42 for the 12-predictor HSI+VBMs+GC-IPL model and was 1.70 for HSI+GC-IPL (Supplementary Table S4).

All seven observed AUCs exceeded their corresponding shuffled-label null distributions in the model-specific permutation analysis, with empirical *p*-values ranging from 0.001 to 0.040. The HSI+GC-IPL model had a permutation *p*-value of 0.001 (Supplementary Figure S3; Supplementary Table S4).

However, paired bootstrap comparisons did not establish statistically significant superiority of HSI+GC-IPL over the alternative models. Compared with HSI alone, the AUC difference was +0.15 (95% CI −0.01 to 0.30; *p* = 0.064), and compared with GC-IPL alone it was +0.11 (95% CI −0.04 to 0.25; *p* = 0.172). The difference relative to HSI+VBMs+GC-IPL was +0.03 (95% CI −0.03 to 0.09; *p* = 0.310). All comparisons against the HSI+GC-IPL model are reported in Supplementary Table S4.

Finally, adding age, sex, and APOE ε4 carrier status did not significantly improve discrimination in the principal models. AUC changed from 0.69 to 0.62 for HSI alone (*p* = 0.204), from 0.73 to 0.75 for GC-IPL alone (*p* = 0.730), and from 0.84 to 0.77 for HSI+GC-IPL (*p* = 0.358) (Supplementary Table S7).

## 4. Discussion

### 4.1. Hyperspectral Imaging Texture Is Associated with Cerebral Amyloid Status

This study provides proof-of-concept evidence that retinal HSI texture features are associated with PET-confirmed cerebral amyloid status in preclinical and prodromal stages of AD. When combined with OCT-derived GC-IPL thickness measures, HSI contributed to the numerically highest classification performance observed in this cohort. However, the improvement over the unimodal models was not statistically resolved, and the findings should therefore be interpreted as exploratory evidence of complementary information rather than definitive multimodal synergy.

Texture-based HSI features showed greater discrimination than normalized spectral reflectance. Lower GLCM COR values were observed in Aβ+ participants, particularly in the inferior macula, indicating greater local variation in neighboring pixel intensities. However, these regional differences were identified within an exploratory biomarker screen and did not remain significant after FDR correction. They should therefore be regarded as candidate signals rather than confirmed regional biomarkers. The inferior hemisphere yielded the highest fixed-feature HSI discrimination, whereas the full macular grid showed slightly lower discrimination but greater longitudinal repeatability. This suggests a possible trade-off between regional sensitivity and measurement robustness. The still moderate repeatability of the full macular grid (ICC = 0.60) may reflect variation in fixation, camera alignment, illumination, ocular media, independently repeated ROI localization, and potentially genuine retinal change between longitudinal measurements. By contrast, reproducibility across different flash settings within the same visit was higher, reaching an ICC of 0.88 for both the full macular grid and inferior hemisphere.

These findings are broadly consistent with previous HSI studies emphasizing the value of spatial as well as spectral information. Lemmens et al. [30] identified discriminative spectral features in patients with established ADD, whereas Hadoux et al. [18] found that raw spectral information alone provided limited separation according to amyloid status and applied dimensionality reduction to reduce non-disease-related spectral variability. Sharafi et al. [19] similarly reported that GLCM-based texture features extracted from perivascular retinal regions contributed to discrimination of likely cerebral amyloid status. Together, these studies suggest that spatial-spectral representations may be more informative than pixel-wise spectral measurements alone in the presence of substantial inter-individual variability [54,55].

The anatomical origin of the observed texture signal remains uncertain. Histopathological studies have reported retinal Aβ in different locations, including peripheral superior-temporal and inferior-temporal regions [14,56,57] as well as the central and perifoveal retina [58]. Retinal Aβ has also been described in association with the vasculature [59,60], raising the possibility that perivascular tissue alterations contribute to HSI texture. From a practical imaging perspective, centrally acquired macular images may also offer greater reproducibility than peripheral retinal imaging, particularly in older participants or individuals with cognitive impairment. GLCM COR quantifies statistical dependence between neighboring gray levels; lower values therefore indicate reduced local intensity correlation and greater texture heterogeneity. The lower GLCM COR observed in Aβ+ participants is qualitatively consistent with ex vivo findings by Zaletel et al. [61], who reported lower GLCM COR in dense-core amyloid plaques than in diffuse plaques. However, the present in vivo retinal signal cannot be attributed directly to retinal amyloid deposits on the basis of these data alone.

The strongest candidate HSI signal occurred at 466 nm in both the full macular grid and inferior hemisphere, corresponding to the shortest wavelength retained by our imaging system. This is biologically compatible with previous experimental and clinical work showing prominent AD-related optical effects at shorter visible wavelengths. More and Vince reported altered short-wavelength retinal intensities in transgenic AD mice before overt plaque formation [15], while subsequent in vivo studies identified prominent spectral differences below approximately 500–565 nm [16,17,18,62,63]. Sharafi et al. [19] likewise reported strong GLCM differences within the approximately 450–550 nm range. Shorter wavelengths are more sensitive to scattering from small-scale tissue structures, providing a plausible optical basis for increased sensitivity in this spectral range.

The wavelength finding also illustrates the potential optimism introduced by data-dependent feature selection. Because 466 nm was identified using the complete cohort, the fixed-feature HSI performance is susceptible to selection-related optimism. When wavelength selection was repeated within each outer cross-validation fold, the full-macula HSI AUC decreased from 0.69 to 0.52. At the same time, 466 nm remained the most frequently selected wavelength, particularly in the inferior hemisphere, where the corresponding reduction in AUC was smaller. These results therefore support 466 nm as a biologically plausible and relatively stable candidate wavelength within this cohort, but not as a prospectively validated feature. Independent studies should evaluate it using a prespecified wavelength and retinal region.

Finally, GLCM COR is an optical texture descriptor rather than a molecular measure, and its pathological specificity remains unknown. In addition to Aβ-related processes, altered retinal texture could reflect phosphorylated tau, neuroinflammation, glial responses, vascular abnormalities, neurodegeneration, or other retinal tissue changes [14,56,64]. Phosphorylated tau has been demonstrated in post-mortem AD retinas [65,66], and hyperspectral imaging studies suggest that both Aβ and tau can produce distinct optical scattering signatures [67]. Moreover, the presence and distribution of retinal Aβ itself remain debated, with both positive and negative histopathological findings reported [68,69,70]. Future work should therefore validate HSI texture features against retinal histopathology or molecular biomarkers, assess their specificity relative to other neurodegenerative and ocular diseases, and determine whether the observed regional and spectral patterns replicate in independent cohorts.

#### Exploratory Non-Monotonic Pattern Across Cross-Sectional AD Stage Groups

Our exploratory cross-sectional analysis revealed a non-monotonic pattern in HSI-derived retinal texture across AD stage groups, with lower GLCM COR in the early Aβ+ group and higher values again in the ADD group. This pattern is consistent with findings from More et al. [16], who reported the largest spectral deviation from controls at the MCI stage, with the discriminative signal becoming less pronounced in more advanced dementia. They proposed that stronger optical effects in earlier disease may relate to small, soluble Aβ assemblies, whereas later stages may involve a greater contribution from larger and more fibrillar aggregates. A similar mechanism could plausibly contribute to the pattern observed here, with greater local texture heterogeneity during earlier amyloid-positive stages and a more optically homogeneous retinal signal at more advanced disease stages.

GLCM COR did not differ significantly between cognitively normal participants from the two independent cohorts, providing some reassurance that the observed stage-group pattern is unlikely to be explained solely by a general cohort-related shift in HSI measurements. Nevertheless, because the ADD participants originated from the additional cohort, disease stage and cohort remain partially confounded, and cohort-specific effects in the ADD group cannot be fully excluded.

Our findings differ from those of Jin et al. [23], who analyzed GLCM features within individual retinal layers on cross-sectional OCT B-scans and reported a more monotonic decline from CN to MCI to AD. This difference may reflect the distinct information captured by the two imaging approaches. En face HSI provides a planar spatial-spectral representation of the retina and may be particularly sensitive to optical and biochemical tissue properties, whereas cross-sectional OCT provides depth-resolved information from specific retinal layers and may capture different structural aspects of disease. The two modalities may therefore exhibit different stage-related patterns.

Overall, the present findings support the hypothesis that retinal HSI texture may vary non-monotonically across the AD continuum. However, because the analysis is cross-sectional, it does not establish within-person temporal changes. Longitudinal studies spanning biomarker-defined disease stages will be important to determine whether this cross-sectional pattern reflects the temporal evolution of retinal optical changes during AD progression.

### 4.2. Macular GC-IPL Measures Suggest Retinal Neurodegenerative Changes

Our structural OCT analysis suggests that the macular GC-IPL may be more sensitive to cerebral amyloid status than the macular RNFL. The joint GC-IPL thickness profile across the nine ETDRS regions was associated with Aβ status in the MANCOVA analysis, whereas the corresponding RNFL profile was not. Importantly, none of the individual GC-IPL regions showed a significant univariate group difference, indicating that the structural signal may be distributed across the spatial thickness pattern rather than driven by a single retinal sector. This is consistent with previous evidence that the macular GC-IPL may be a sensitive marker of AD-related neurodegeneration, potentially owing to its high ganglion-cell density and relatively low inter-individual variability [22]. Macular thinning has also been reported in cognitively unimpaired individuals with risk factors for AD, including APOE ε4 carriage and family history [71]. Moreover, GC-IPL thinning has been associated with higher p-tau burden [72], and thinner baseline GC-IPL has been linked to an increased risk of subsequent cognitive decline [73]. A recent systematic review of OCT findings in early AD likewise identified GC-IPL abnormalities, including thinning, as a recurrent finding, while emphasizing substantial heterogeneity in disease stage, OCT methodology, segmentation procedures, and confounder control [74].

At the same time, evidence from Jali et al. highlights an important limitation of global retinal thickness measures. Although they observed progressive GC-IPL and RNFL thinning across the continuum from normal cognition to dementia, average GC-IPL (AUC = 0.60) and RNFL (AUC = 0.56) showed limited discriminatory utility for distinguishing individuals with normal cognition or MCI from those at more advanced disease stages [75]. This limited performance may partly reflect non-uniform retinal thickness changes in AD, as previous studies have reported both thinning and thickening of inner retinal layers, potentially reflecting differences in disease stage or glial responses [76,77]. Such spatial heterogeneity may be obscured when thickness is averaged over large retinal areas.

Consistent with this interpretation, the classifier using all nine GC-IPL regions achieved an AUC of 0.73, whereas a model based on the global mean of those same nine sectors performed approximately at chance level (AUC = 0.45). The reduced-complexity sensitivity analyses further supported the usefulness of retaining spatially resolved information. Elastic-net regularization produced nearly identical discrimination to the original nine-region model (AUC = 0.73 in both models), whereas fold-internal PCA reduced the number of effective predictors to approximately four components but also reduced discrimination to an AUC of 0.61. Although the differences between these models were not statistically significant, the results are consistent with the possibility that aggressive dimensionality reduction removes spatial information that contributes to classification. At the same time, the low EPV of the nine-region model warrants caution, and the apparent benefit of spatially resolved GC-IPL measurements requires validation in larger cohorts.

We also observed an inferior-predominant regional pattern. The inferior ETDRS regions (II and IO) yielded an AUC of 0.71, whereas the corresponding superior regions (SI and SO) yielded an AUC of 0.45. A similar inferior-predominant pattern was observed for HSI, with both modalities showing stronger discrimination in inferior rather than superior macular regions. Because HSI and OCT were evaluated in the same participants, this represents within-cohort multimodal convergence rather than independent replication. Nevertheless, the concordant regional pattern across two distinct imaging modalities is of interest and provides a hypothesis for future anatomically targeted studies. These patterns also show a possible anatomical resemblance to those observed in glaucoma, although this should not be interpreted as evidence of a shared mechanism; the differential diagnosis between AD-related and glaucomatous retinal changes remains unresolved [78,79].

In our cohort, age was significantly associated with macular RNFL but not GC-IPL thickness, while sex was significantly associated with GC-IPL but not RNFL. This pattern is somewhat unexpected, as previous studies generally report both age- and sex-related differences across several macular layers [80,81]. Overall, the literature indicates that retinal thickness measures are influenced by demographic factors, underscoring the importance of accounting for these variables. In our classification sensitivity analyses, adding age, sex, and APOE ε4 status did not significantly improve the GC-IPL model. The divergent associations observed in the MANCOVA analysis may therefore reflect cohort-specific variation rather than a true biological absence of age or sex effects.

Beyond conventional thickness measures, OCT images can also be analyzed using spatial and texture information. Jin et al. [23] recently reported that OCT-based GLCM features outperformed average thickness measures in distinguishing AD and MCI from controls. Similarly, emerging DL approaches that process complete GC-IPL or RNFL thickness maps directly, rather than relying on summary measures, have shown promising results in AD and MCI classification [82]. These developments further support the potential value of preserving spatial retinal information. However, the lack of large, publicly available OCT datasets remains an important limitation for such approaches, with most studies based on relatively small, private cohorts collected across heterogeneous clinical settings [83].

### 4.3. Limited Contribution of CFP-Derived Vascular Biomarkers

Our comprehensive analysis of 24 VBMs revealed limited evidence for differences according to cerebral amyloid status. CRAE and venous endpoint count showed nominal group differences, but neither remained significant after Benjamini–Hochberg FDR correction. Accordingly, these individual vascular associations should be regarded as exploratory candidate signals rather than confirmed amyloid-associated vascular changes. When used for classification, CRAE and CRVE yielded modest discrimination (AUC = 0.68; permutation *p* = 0.040). Our findings are nevertheless broadly consistent with Sharafi et al. [19], who also reported wider arteriolar diameters in Aβ+ participants, suggesting that retinal vascular alterations may accompany biochemical and structural changes. Conversely, a meta-analysis pooling results from four studies found no significant association between CRAE or CRVE and cognitive impairment [84]. That analysis showed substantial between-study heterogeneity (I^2^ = 93%), which may reflect differences in disease stage, study design, imaging protocols, vascular measurement techniques, and participant characteristics.

One possible explanation for the limited contribution observed here is that the CFP-derived measures primarily characterize static large-vessel morphology. More recent evidence suggests that AD-related vascular abnormalities may be more apparent at the capillary and perfusion level. A recent meta-analysis of 36 OCTA studies involving more than 4000 participants reported reduced retinal vascular density in both AD and MCI [85], while a recent OCT/OCTA study identified additional capillary abnormalities in early AD and MCI [86]. These findings suggest that capillary-level measures may be more sensitive to early AD-related vascular dysfunction than static CRAE/CRVE measurements.

This interpretation is also consistent with the functional vascular literature. Querques et al. [87], using OCTA and dynamic vessel analysis (DVA), found impaired flicker-induced vascular reactivity in individuals with MCI despite limited structural vessel differences, suggesting that functional alterations may precede overt morphological changes. Functional vascular imaging such as DVA may therefore offer greater sensitivity to early disease-related vascular dysfunction. In this context, DVA data are currently being collected in the ongoing RetAD trial and will be analyzed in future work.

Retinal vessel caliber is also biologically non-specific and is influenced by systemic vascular and metabolic factors, including hypertension, diabetes, and cardiovascular disease [88]. This may increase inter-individual variability and reduce the specificity of CRAE and CRVE for cerebral amyloid status.

Methodological factors should also be considered. The present cohort is small, and the HSI+GC-IPL model already contains 10 predictors for 17 Aβ+ events (EPV = 1.70). Adding CRAE and CRVE increases the model to 12 predictors and reduces the EPV to 1.42. Consistent with this, adding CRAE and CRVE to HSI+GC-IPL did not improve discrimination in this cohort; the observed AUC decreased from 0.84 to 0.81. We therefore interpret the contribution of these specific CFP-derived vascular measures as limited rather than absent, and we cannot conclude that vascular information is redundant with the other modalities.

Overall, the present findings support further investigation of retinal vascular biomarkers using more sensitive capillary-level and functional approaches, including OCTA and DVA, in larger biomarker-confirmed cohorts.

### 4.4. Multimodal Retinal Imaging Maps onto the ATV(N) Framework

The AT(N) framework classifies AD biomarkers according to the pathological processes they reflect: Aβ deposition (A), pathologic tau (T), and neurodegeneration (N) [89]. From this perspective, our multimodal retinal approach may capture complementary retinal correlates of several of these processes. HSI-derived texture changes were associated with cerebral amyloid status in the present study and may therefore reflect biochemical alterations related to Aβ pathology (A), whereas previous work has shown that hyperspectral signatures can also be sensitive to phosphorylated tau (T) [67]. OCT-derived GC-IPL thickness, in turn, provides a structural measure of retinal neurodegeneration and may represent an N-related component. This provides a biologically plausible framework for understanding why HSI and OCT may offer complementary information.

The vascular component may provide an additional dimension. The 2024 revised criteria for AD diagnosis and staging recognize vascular brain injury (V) as an important non-AD co-pathology alongside the core AD processes [2]. Although the CFP-derived vascular biomarkers evaluated here should not be considered direct equivalents of cerebral V biomarkers, they may capture retinal manifestations of vascular dysfunction that interact with AD pathology. This possibility is supported by evidence that vascular burden can accelerate cognitive decline, particularly in individuals with early Alzheimer’s pathological changes [90], and by broader work implicating vascular dysregulation early in the development of late-onset AD [91]. The limited incremental contribution of CRAE and CRVE in the present cohort therefore does not exclude a role for vascular retinal biomarkers, particularly if more sensitive capillary-level or functional measures are used.

Within this framework, multimodal retinal imaging can be viewed as a potential means of jointly sampling biochemical, structural, and vascular aspects of retinal involvement in AD, conceptually paralleling the A, T, N, and V dimensions used in contemporary biomarker frameworks. However, this mapping should be regarded as hypothesis-generating rather than as evidence that the retinal measures directly quantify the corresponding cerebral pathological processes. The numerically higher performance of the HSI+GC-IPL model is compatible with this interpretation, although confirmation in larger cohorts is required. Future studies integrating retinal imaging with established amyloid, tau, neurodegeneration, and vascular biomarkers will be needed to determine how closely these retinal signals correspond to specific AT(V)N processes.

### 4.5. Strengths and Limitations

A key strength of this study is the use of a well-characterized cohort spanning the preclinical and prodromal stages of AD, with cerebral amyloid status established using PET rather than clinical diagnosis alone. The multimodal design enabled biochemical or optical, structural, and vascular retinal information to be evaluated within the same participants and study eye. In addition, the snapshot HSI system allows rapid image acquisition, reducing susceptibility to motion artifacts and supporting potential clinical scalability [36]. Measurement stability of the HSI texture features was evaluated using repeatability and reproducibility analyses, and the classification framework was supplemented with permutation testing, bootstrap confidence intervals, paired model comparisons, and sensitivity analyses addressing feature selection, model complexity, confounding, and GC-IPL dimensionality.

The principal limitation is the modest sample size of the primary cohort (N = 40; 17 Aβ+), particularly relative to the number of predictors in several multimodal models. The resulting low events-per-variable values and wide AUC confidence intervals indicate substantial uncertainty in model stability and increase the risk of overfitting. Moreover, the HSI feature carried forward into the multimodal models was identified through exploratory analysis of the same cohort. Although fold-internal wavelength selection was performed as a sensitivity analysis, the reduction in full-macula HSI performance indicates selection-related optimism in the fixed-feature estimate. Likewise, none of the individual exploratory retinal biomarker comparisons remained significant after FDR correction. The reported classification results should therefore be regarded as internally cross-validated, hypothesis-generating estimates rather than validated measures of clinical performance. No independent external validation cohort with equivalent PET-confirmed Aβ status was available, and prospective validation with prespecified retinal regions and HSI features is required.

Several additional limitations affect biological interpretation and generalizability. The study excluded participants with other major neurodegenerative and common ocular diseases, including glaucoma, and therefore does not establish the disease specificity of the identified retinal signals. GLCM COR is an optical texture descriptor rather than a molecular measure, GC-IPL thickness is not specific to AD-related neurodegeneration, and retinal vascular measures are influenced by systemic vascular and metabolic factors. The exploratory disease-stage analysis was also cross-sectional and combined participants from two separately recruited cohorts, leaving disease stage partially confounded with cohort despite similar HSI measurements between the cognitively normal groups. Finally, HSI repeatability across longitudinal visits was moderate, and residual variability related to image acquisition, illumination, fixation, ocular media, and manual foveal localization cannot be excluded. Larger, longitudinal, independently recruited cohorts including relevant neurological and ocular comparison groups will therefore be necessary to establish robustness, pathological specificity, and clinical utility.

## 5. Conclusions

Multimodal retinal imaging combining HSI-derived texture features and OCT-based structural measurements shows promise for identifying retinal signals associated with early cerebral amyloid pathology. In this proof-of-concept cohort, the HSI+GC-IPL model achieved the highest observed discrimination, suggesting that combining optical and structural retinal information may improve early AD characterization. These findings require validation in larger, independent, and longitudinal cohorts with prespecified retinal features and relevant neurological and ocular comparison groups. If confirmed, multimodal retinal imaging could offer a scalable and accessible approach for early AD risk stratification and clinical-trial enrichment using existing ophthalmic infrastructure.

## Figures and Tables

**Table 1:** Demographic characteristics and cognitive scores of Aβ− and Aβ+ participants in the CN/MCI cohort.

	Aβ− (N = 23) CN	Aβ+ (N = 17) CN	Aβ+ (N = 17) MCI	*p*-Value
Total Participants	23	10	7	-
Sex (M/F)	9/14	3/7	2/5	0.815 ‡
Age (yrs: Mean ± SD)	76.2 ± 7.1	76.2 ± 3.9	70.3 ± 10.4	0.284 †
MMSE (Mean ± SD)	29.0 ± 1.1	29.2 ± 0.8	21.1 ± 5.2	0.000 †
APOE genotype, n (%)				0.052 ‡
ε2/ε2	0 (0.0)	0 (0.0)	0 (0.0)	
ε2/ε3	0 (0.0)	0 (0.0)	0 (0.0)	
ε3/ε3	12 (52.2)	4 (40.0)	1 (14.3)	
ε2/ε4	1 (4.3)	0 (0.0)	0 (0.0)	
ε3/ε4	10 (43.5)	6 (60.0)	4 (57.1)	
ε4/ε4	0 (0.0)	0 (0.0)	2 (28.6)	
AVLT total learning (Mean ± SD) *	47.0 ± 9.9	47.6 ± 7.3	19.1 ± 6.8	0.000 †
BNT *	55.2 ± 3.3	55.2 ± 3.5	45.6 ± 5.9	0.004 †
AVF *	20.0 ± 5.7	17.5 ± 6.7	12.9 ± 2.3	0.008 †

Aβ− = amyloid-beta negative; Aβ+ = amyloid-beta positive; SD = standard deviation; M = male; F = female; CN = cognitively normal; MCI = mild cognitive impairment; MMSE = mini-mental state examination; APOE = apolipoprotein E; yrs = years; AVLT = auditory verbal learning test; BNT = Boston naming test; AVF = Animal Verbal Fluency; ‡ Chi-square test; † Kruskal–Wallis test; * No measurements for 5 CN subjects, see Supplementary File S2.

**Table 2:** HSI classification performance, repeatability, and reproducibility by macular grid.

Macular Region	AUC (95% CI)	Sensitivity	Specificity	Repeatability ICC	Reproducibility ICC
Full Macular Grid	0.69 (0.50–0.85)	**0.71**	0.70	**0.60**	**0.88**
Superior Hemisphere	0.62 (0.43–0.79)	0.47	0.61	0.48	0.64
Inferior Hemisphere	**0.72 (0.54–0.87)**	0.65	**0.74**	0.51	**0.88**

The highest value per performance metric within each column is highlighted in bold. HSI = hyperspectral imaging; AUC = area under the curve; CI = confidence interval; ICC = intraclass correlation coefficient.

**Table 3:** MANCOVA results for retinal thickness measures.

OCT Layer	Aβ Status (*p*)	Sex (*p*)	Age (*p*)
GC-IPL	**0.009**	**0.041**	0.600
RNFL	0.694	0.059	**0.024**

Significant *p*-values are highlighted in bold. MANCOVA = multivariate analysis of covariance; OCT = optical coherence tomography; GC-IPL = ganglion cell–inner plexiform layer; RNFL = retinal nerve fiber layer; Aβ = amyloid-beta.

**Table 4:** OCT-based classification performance using GC-IPL thickness measures.

GC-IPL Features	AUC (95% CI)	Sensitivity	Specificity
9 ETDRS regions	**0.73 (0.56–0.88)**	**0.53**	**0.65**
Superior (SI, SO)	0.45 (0.25–0.65)	0.41	0.44
Inferior (II, IO)	0.71 (0.54–0.87)	**0.53**	0.61

The highest value per performance metric within each column is highlighted in bold. OCT = optical coherence tomography; GC-IPL = ganglion cell–inner plexiform layer; ETDRS = Early Treatment Diabetic Retinopathy Study; SI = superior inner; SO = superior outer; II = inferior inner; IO = inferior outer; AUC = area under the receiver operating characteristic curve; CI = confidence interval.

**Table 5:** VBM-based classification performance.

VBMs	AUC (95% CI)	Sensitivity	Specificity
CRAE + CRVE	**0.68 (0.50–0.84)**	**0.65**	**0.65**
CRAE	0.66 (0.48–0.83)	0.59	0.61
CRVE	0.48 (0.27–0.67)	0.47	0.48

The highest value per performance metric within each column is highlighted in bold. VBM = vascular biomarker; CRAE = Central Retinal Arteriolar Equivalent; CRVE = Central Retinal Venular Equivalent; AUC = area under the receiver operating characteristic curve; CI = confidence interval.

## Data Availability

The datasets analyzed during the current study are available from the corresponding author on reasonable request. The code used for statistical analysis and classification will be made publicly available in an online repository upon publication.

## Supplementary Materials

The following supporting information can be downloaded at https://www.mdpi.com/article/10.3390/bioengineering13091041/s1. File S1: Supplementary Methods: fold-internal HSI wavelength-selection sensitivity analysis, GC-IPL dimensionality-reduction sensitivity analyses, and covariate-augmented sensitivity analyses; Supplementary Figure S1: spectral reflectance and texture features across macular grids by amyloid status; Supplementary Figure S2: GC-IPL thickness by ETDRS region and amyloid status; Supplementary Figure S3: permutation test (observed AUC versus its model-specific null distribution) for the seven primary classification models; Supplementary Figure S4: sensitivity of HSI classification performance to the wavelength-selection procedure, and stability of fold-internal wavelength selection, by macular region; Supplementary Figure S5: RNFL thickness by ETDRS region and amyloid status; Supplementary Figure S6: repeat-visit macular grid alignment; Supplementary Figure S7: GLCM correlation (466 nm, full macular grid) across four cross-sectional disease-stage groups; Supplementary Table S1: demographic characteristics of the combined cohorts; Supplementary Table S2: summary of CFP-derived retinal vascular biomarkers by amyloid status; Supplementary Table S3: classification performance for unimodal and multimodal feature sets across macular regions; Supplementary Table S4: classification performance, events per predictor variable, permutation *p*-values, and paired-bootstrap ΔAUC comparisons against the HSI+GC-IPL model for the seven primary models; Supplementary Table S5: fold-internal wavelength-selection stability by macular region; Supplementary Table S6: GC-IPL dimensionality-reduction sensitivity models using elastic-net regularization, fold-internal PCA, and a global-mean GC-IPL feature; Supplementary Table S7: covariate-augmented sensitivity models including age, sex, and APOE ε4 carrier status; Supplementary Table S8: Benjamini–Hochberg FDR-adjusted q-values by feature family; Supplementary Table S9: RNFL descriptive statistics by ETDRS region and amyloid status; Supplementary Table S10: HSI GLCM correlation nominal *p*-values and Benjamini–Hochberg-adjusted q-values by wavelength and macular region; Supplementary Table S11: HSI normalized-reflectance nominal *p*-values and Benjamini–Hochberg-adjusted q-values by wavelength and macular region; Supplementary Table S12: GC-IPL descriptive statistics by ETDRS region and amyloid status. Supplementary File S2: Excel file containing detailed demographic information and cognitive test scores for individual participants.

## References

[1] Nichols E., Steinmetz J.D., Vollset S.E., Fukutaki K., Chalek J., Abd-Allah F., Abdoli A., Abualhasan A., Abu-Gharbieh E., Akram T.T. (2022). Estimation of the Global Prevalence of Dementia in 2019 and Forecasted Prevalence in 2050: An Analysis for the Global Burden of Disease Study 2019. Lancet Public Health.

[2] Jack C.R., Andrews J.S., Beach T.G., Buracchio T., Dunn B., Graf A., Hansson O., Ho C., Jagust W., McDade E. (2024). Revised Criteria for Diagnosis and Staging of Alzheimer’s Disease: Alzheimer’s Association Workgroup. Alzheimer’s Dement..

[3] Greenberg S.M., Bacskai B.J., Hernandez-Guillamon M., Pruzin J., Sperling R., van Veluw S.J. (2020). Cerebral Amyloid Angiopathy and Alzheimer Disease—One Peptide, Two Pathways. Nat. Rev. Neurol..

[4] Gustavsson A., Norton N., Fast T., Frölich L., Georges J., Holzapfel D., Kirabali T., Krolak-Salmon P., Rossini P.M., Ferretti M.T. (2023). Global Estimates on the Number of Persons Across the Alzheimer’s Disease Continuum. Alzheimer’s Dement..

[5] van Dyck C.H., Swanson C.J., Aisen P., Bateman R.J., Chen C., Gee M., Kanekiyo M., Li D., Reyderman L., Cohen S. (2023). Lecanemab in Early Alzheimer’s Disease. N. Engl. J. Med..

[6] Livingston G., Huntley J., Liu K.Y., Costafreda S.G., Selbæk G., Alladi S., Ames D., Banerjee S., Burns A., Brayne C. (2024). Dementia Prevention, Intervention, and Care: 2024 Report of the Lancet Standing Commission. Lancet.

[7] Cummings J.L., Zhou Y., Lee G., Zhong K., Fonseca J., Leisgang-Osse A.M., Cheng F. (2025). Alzheimer’s Disease Drug Development Pipeline: 2025. Alzheimer’s Dement. Transl. Res. Clin. Interv..

[8] Bilgel M., An Y., Walker K.A., Moghekar A.R., Ashton N.J., Kac P.R., Karikari T.K., Blennow K., Zetterberg H., Jedynak B.M. (2023). Longitudinal Changes in Alzheimer’s-Related Plasma Biomarkers and Brain Amyloid. Alzheimer’s Dement..

[9] Palmqvist S., Zetterberg H., Mattsson N., Johansson P., Minthon L., Blennow K., Olsson M., Hansson O., Alzheimer’s Disease Neuroimaging Initiative, Swedish BioFINDER Study Group (2015). Detailed Comparison of Amyloid PET and CSF Biomarkers for Identifying Early Alzheimer Disease. Neurology.

[10] Cummings J., Aisen P.S., DuBois B., Frölich L., Jack C.R., Jones R.W., Morris J.C., Raskin J., Dowsett S.A., Scheltens P. (2016). Drug Development in Alzheimer’s Disease: The Path to 2025. Alzheimer’s Res. Ther..

[11] Hansson O., Blennow K., Zetterberg H., Dage J. (2023). Blood Biomarkers for Alzheimer’s Disease in Clinical Practice and Trials. Nat. Aging.

[12] London A., Benhar I., Schwartz M. (2013). The Retina as a Window to the Brain—From Eye Research to CNS Disorders. Nat. Rev. Neurol..

[13] Christinaki E., Kulenovic H., Hadoux X., Baldassini N., Van Eijgen J., De Groef L., Stalmans I., van Wijngaarden P. (2022). Retinal Imaging Biomarkers of Neurodegenerative Diseases. Clin. Exp. Optom..

[14] Gaire B.P., Koronyo Y., Fuchs D.-T., Shi H., Rentsendorj A., Danziger R., Vit J.-P., Mirzaei N., Doustar J., Sheyn J. (2024). Alzheimer’s Disease Pathophysiology in the Retina. Prog. Retin. Eye Res..

[15] More S.S., Vince R. (2015). Hyperspectral Imaging Signatures Detect Amyloidopathy in Alzheimer’s Mouse Retina Well Before Onset of Cognitive Decline. ACS Chem. Neurosci..

[16] More S.S., Beach J.M., McClelland C., Mokhtarzadeh A., Vince R. (2019). In Vivo Assessment of Retinal Biomarkers by Hyperspectral Imaging: Early Detection of Alzheimer’s Disease. ACS Chem. Neurosci..

[17] Vandenabeele M., Veys L., Lemmens S., Hadoux X., Gelders G., Masin L., Serneels L., Theunis J., Saito T., Saido T.C. (2021). The AppNL-G-F Mouse Retina Is a Site for Preclinical Alzheimer’s Disease Diagnosis and Research. Acta Neuropathol. Commun..

[18] Hadoux X., Hui F., Lim J.K.H., Masters C.L., Pébay A., Chevalier S., Ha J., Loi S., Fowler C.J., Rowe C. (2019). Non-Invasive In Vivo Hyperspectral Imaging of the Retina for Potential Biomarker Use in Alzheimer’s Disease. Nat. Commun..

[19] Sharafi S.M., Sylvestre J.-P., Chevrefils C., Soucy J.-P., Beaulieu S., Pascoal T.A., Arbour J.D., Rhéaume M.-A., Robillard A., Chayer C. (2019). Vascular Retinal Biomarkers Improves the Detection of the Likely Cerebral Amyloid Status from Hyperspectral Retinal Images. Alzheimer’s Dement. Transl. Res. Clin. Interv..

[20] Byun M.S., Park S.W., Lee J.H., Yi D., Jeon S.Y., Choi H.J., Joung H., Ghim U.H., Park U.C., Kim Y.K. (2021). Association of Retinal Changes with Alzheimer Disease Neuroimaging Biomarkers in Cognitively Normal Individuals. JAMA Ophthalmol..

[21] Almeida A.L.M., Pires L.A., Figueiredo E.A., Costa-Cunha L.V.F., Zacharias L.C., Preti R.C., Monteiro M.L.R., Cunha L.P. (2019). Correlation Between Cognitive Impairment and Retinal Neural Loss Assessed by Swept-Source Optical Coherence Tomography in Patients with Mild Cognitive Impairment. Alzheimer’s Dement..

[22] Chan V.T.T., Sun Z., Tang S., Chen L.J., Wong A., Tham C.C., Wong T.Y., Chen C., Ikram M.K., Whitson H.E. (2019). Spectral-Domain OCT Measurements in Alzheimer’s Disease: A Systematic Review and Meta-Analysis. Ophthalmology.

[23] Jin Z., Wang X., Lang Y., Song Y., Zhan H., Shama W., Shen Y., Zeng G., Zhou F., Gao H. (2025). Retinal Optical Coherence Tomography Intensity Spatial Correlation Features as New Biomarkers for Confirmed Alzheimer’s Disease. Alzheimer’s Res. Ther..

[24] Liu Y.-L., Hsieh Y.-T., Chen T.-F., Chiou J.-M., Tsai M.-K., Chen J.-H., Chen Y.-C. (2018). Retinal Ganglion Cell–Inner Plexiform Layer Thickness Is Nonlinearly Associated with Cognitive Impairment in the Community-Dwelling Elderly. Alzheimer’s Dement..

[25] Rebouças S.C.L., Cougnard-Gregoire A., Arnould L., Delyfer M.-N., Schweitzer C., Korobelnik J.-F., Foubert-Samier A., Cheung C.Y., Wong T.Y., Delcourt C. (2023). Retinal Microvasculature and Incident Dementia over 10 Years: The Three-City-Alienor Cohort. Alzheimer’s Dement..

[26] Bahr T., Vu T.A., Tuttle J.J., Iezzi R. (2024). Deep Learning and Machine Learning Algorithms for Retinal Image Analysis in Neurodegenerative Disease: Systematic Review of Datasets and Models. Transl. Vis. Sci. Technol..

[27] Mohsen S. (2025). Alzheimer’s Disease Detection Using Deep Learning and Machine Learning: A Review. Artif. Intell. Rev..

[28] Fhima J., Van Eijgen J., Billen Moulin-Romsée M.I., Brackenier H., Kulenovic H., Debeuf V., Vangilbergen M., Freiman M., Stalmans I., Behar J.A. (2024). LUNet: Deep Learning for the Segmentation of Arterioles and Venules in High Resolution Fundus Images. Physiol. Meas..

[29] Fhima J., Van Eijgen J., Stalmans I., Men Y., Freiman M., Behar J.A., Karlinsky L., Michaeli T., Nishino K. (2023). PVBM: A Python Vasculature Biomarker Toolbox Based on Retinal Blood Vessel Segmentation. Computer Vision—ECCV 2022 Workshops.

[30] Lemmens S., Van Craenendonck T., Van Eijgen J., De Groef L., Bruffaerts R., Andrade de Jesus D., Charle W., Jayapala M., Sunaric-Mégevand G., Standaert A. (2020). Combination of Snapshot Hyperspectral Retinal Imaging and Optical Coherence Tomography to Identify Alzheimer’s Disease Patients. Alzheimer’s Res. Ther..

[31] Zhang R., Sheng J., Zhang Q., Wang J., Wang B. (2025). A Review of Multimodal Fusion-Based Deep Learning for Alzheimer’s Disease. Neuroscience.

[32] Schaeverbeke J.M., Gabel S., Meersmans K., Luckett E.S., De Meyer S., Adamczuk K., Nelissen N., Goovaerts V., Radwan A., Sunaert S. (2021). Baseline Cognition Is the Best Predictor of 4-Year Cognitive Change in Cognitively Intact Older Adults. Alzheimer’s Res. Ther..

[33] Klunk W.E., Koeppe R.A., Price J.C., Benzinger T.L., Devous M.D., Jagust W.J., Johnson K.A., Mathis C.A., Minhas D., Pontecorvo M.J. (2015). The Centiloid Project: Standardizing Quantitative Amyloid Plaque Estimation by PET. Alzheimer’s Dement..

[34] Adamczuk K., De Weer A.S., Nelissen N., Chen K., Sleegers K., Bettens K., Van Broeckhoven C., Vandenbulcke M., Thiyyagura P., Dupont P. (2013). Polymorphism of Brain Derived Neurotrophic Factor Influences β Amyloid Load in Cognitively Intact Apolipoprotein E ε4 Carriers. NeuroImage Clin..

[35] Lambrechts A., Gonzalez P., Geelen B., Soussan P., Tack K., Jayapala M. A CMOS-Compatible, Integrated Approach to Hyper- and Multispectral Imaging. Proceedings of the 2014 IEEE International Electron Devices Meeting (IEDM).

[36] Geelen B., Tack N., Lambrechts A., von Freymann G., Schoenfeld W.V., Rumpf R.C. (2014). A Compact Snapshot Multispectral Imager with a Monolithically Integrated Per-Pixel Filter Mosaic. Advanced Fabrication Technologies for Micro/Nano Optics and Photonics VII.

[37] Pichette J., Goossens T., Vunckx K., Lambrechts A., Soskind Y.G., Olson C. (2017). Hyperspectral Calibration Method for CMOS-Based Hyperspectral Sensors. Photonic Instrumentation Engineering IV.

[38] Giancardo L., Meriaudeau F., Karnowski T.P., Chaum E., Tobin K., Campolo D. (2010). Quality Assessment of Retinal Fundus Images Using Elliptical Local Vessel Density. New Developments in Biomedical Engineering.

[39] (1991). Early Treatment Diabetic Retinopathy Study Design and Baseline Patient Characteristics: ETDRS Report Number 7. Ophthalmology.

[40] Haralick R.M., Shanmugam K., Dinstein I. (1973). Textural Features for Image Classification. IEEE Trans. Syst. Man Cybern..

[41] Fhima J., Van Eijgen J., Reiner-Benaim A., Beeckmans L., Abramovich O., Stalmans I., Behar J.A. (2025). Computerized Analysis of the Eye Vasculature in a Mass Dataset of Digital Fundus Images: The Example of Age, Sex, and Primary Open-Angle Glaucoma. Ophthalmol. Sci..

[42] Knudtson M.D., Lee K.E., Hubbard L.D., Wong T.Y., Klein R., Klein B.E.K. (2003). Revised Formulas for Summarizing Retinal Vessel Diameters. Curr. Eye Res..

[43] Bender R., Lange S. (2001). Adjusting for Multiple Testing—When and How?. J. Clin. Epidemiol..

[44] Benjamini Y., Hochberg Y. (1995). Controlling the False Discovery Rate: A Practical and Powerful Approach to Multiple Testing. J. R. Stat. Soc. Ser. B.

[45] Fawcett T. (2006). An Introduction to ROC Analysis. Pattern Recognit. Lett..

[46] Sokolova M., Lapalme G. (2009). A Systematic Analysis of Performance Measures for Classification Tasks. Inf. Process. Manag..

[47] Van Calster B., Collins G.S., Vickers A.J., Wynants L., Kerr K.F., Barreñada L., Varoquaux G., Singh K., Moons K.G.M., Hernandez-Boussard T. (2025). Evaluation of Performance Measures in Predictive Artificial Intelligence Models to Support Medical Decisions: Overview and Guidance. Lancet Digit. Health.

[48] Peduzzi P., Concato J., Kemper E., Holford T.R., Feinstein A.R. (1996). A Simulation Study of the Number of Events per Variable in Logistic Regression Analysis. J. Clin. Epidemiol..

[49] van Smeden M., de Groot J.A.H., Moons K.G.M., Collins G.S., Altman D.G., Eijkemans M.J.C., Reitsma J.B. (2016). No Rationale for 1 Variable per 10 Events Criterion for Binary Logistic Regression Analysis. BMC Med. Res. Methodol..

[50] Riley R.D., Ensor J., Snell K.I.E., Harrell F.E., Martin G.P., Reitsma J.B., Moons K.G.M., Collins G., van Smeden M. (2020). Calculating the Sample Size Required for Developing a Clinical Prediction Model. BMJ.

[51] Ojala M., Garriga G.C. (2010). Permutation Tests for Studying Classifier Performance. J. Mach. Learn. Res..

[52] Phipson B., Smyth G.K. (2010). Permutation P-values Should Never Be Zero: Calculating Exact P-values When Permutations Are Randomly Drawn. Stat. Appl. Genet. Mol. Biol..

[53] Varma S., Simon R. (2006). Bias in Error Estimation When Using Cross-Validation for Model Selection. BMC Bioinform..

[54] Kumar B., Dikshit O., Gupta A., Singh M.K. (2020). Feature Extraction for Hyperspectral Image Classification: A Review. Int. J. Remote Sens..

[55] He L., Li J., Liu C., Li S. (2018). Recent Advances on Spectral–Spatial Hyperspectral Image Classification: An Overview and New Guidelines. IEEE Trans. Geosci. Remote Sens..

[56] Koronyo Y., Rentsendorj A., Mirzaei N., Regis G.C., Sheyn J., Shi H., Barron E., Cook-Wiens G., Rodriguez A.R., Medeiros R. (2023). Retinal Pathological Features and Proteome Signatures of Alzheimer’s Disease. Acta Neuropathol..

[57] Koronyo Y., Biggs D., Barron E., Boyer D.S., Pearlman J.A., Au W.J., Kile S.J., Blanco A., Fuchs D.T., Ashfaq A. (2017). Retinal Amyloid Pathology and Proof-of-Concept Imaging Trial in Alzheimer’s Disease. JCI Insight.

[58] Lee S., Jiang K., McIlmoyle B., To E., Xu Q., Hirsch-Reinshagen V., Mackenzie I.R., Hsiung G.Y.R., Eadie B.D., Sarunic M.V. (2020). Amyloid Beta Immunoreactivity in the Retinal Ganglion Cell Layer of the Alzheimer’s Eye. Front. Neurosci..

[59] Gonzales M., Fuchs D.T., Doustar J., Koronyo Y., Sherman D.S., Lyden P.D., Sicotte N.L., Black K.L., Kremen S., Dumitrascu O.M. (2025). Integrated Analysis of In Vivo Retinal Perivascular Amyloid Imaging and Cognition. Alzheimer’s Dement..

[60] Dumitrascu O.M., Doustar J., Fuchs D.T., Koronyo Y., Sherman D.S., Miller M.S., Johnson K.O., Carare R.O., Verdooner S.R., Lyden P.D. (2024). Retinal Peri-Arteriolar Versus Peri-Venular Amyloidosis, Hippocampal Atrophy, and Cognitive Impairment: Exploratory Trial. Acta Neuropathol. Commun..

[61] Zaletel I., Milutinović K., Bajčetić M., Nowakowski R.S. (2021). Differentiation of Amyloid Plaques Between Alzheimer’s Disease and Non-Alzheimer’s Disease Individuals Based on Gray-Level Co-Occurrence Matrix Texture Analysis. Microsc. Microanal..

[62] More S.S., Beach J.M., Vince R. (2016). Early Detection of Amyloidopathy in Alzheimer’s Mice by Hyperspectral Endoscopy. Investig. Ophthalmol. Vis. Sci..

[63] Oba R., Ujike N., Ono Y., Okano T., Murakami T. (2024). Label-Free Autofluorescence and Hyperspectral Imaging of Cerebral Amyloid-β Lesions in Aged Squirrel Monkeys. J. Vet. Diagn. Investig..

[64] Salajková Z., Barolo L., Baiocco P., Ruzicka B., Mura F., Di Lorenzo F., Boffi A., Ricco V., Ruocco G., Leonetti M. (2025). Optical Signature of Retinal Tau Fibrillation. Sci. Rep..

[65] Davis M.R., Robinson E., Koronyo Y., Salobrar-Garcia E., Rentsendorj A., Gaire B.P., Mirzaei N., Kayed R., Sadun A.A., Ljubimov A.V. (2025). Retinal Ganglion Cell Vulnerability to Pathogenic Tau in Alzheimer’s Disease. Acta Neuropathol. Commun..

[66] Walkiewicz G., Ronisz A., Van Ginderdeuren R., Lemmens S., Bouwman F.H., Hoozemans J.J.M., Morrema T.H.J., Rozemuller A.J., Hart de Ruyter F.J., De Groef L. (2024). Primary Retinal Tauopathy: A Tauopathy with a Distinct Molecular Pattern. Alzheimer’s Dement..

[67] Du X., Koronyo Y., Mirzaei N., Yang C., Fuchs D.T., Black K.L., Koronyo-Hamaoui M., Gao L. (2022). Label-Free Hyperspectral Imaging and Deep-Learning Prediction of Retinal Amyloid β-Protein and Phosphorylated Tau. PNAS Nexus.

[68] Williams E.A., McGuone D., Frosch M.P., Hyman B.T., Laver N., Stemmer-Rachamimov A. (2017). Absence of Alzheimer Disease Neuropathologic Changes in Eyes of Subjects with Alzheimer Disease. J. Neuropathol. Exp. Neurol..

[69] Schön C., Hoffmann N.A., Ochs S.M., Burgold S., Filser S., Steinbach S., Seeliger M.W., Arzberger T., Goedert M., Kretzschmar H.A. (2012). Long-Term In Vivo Imaging of Fibrillar Tau in the Retina of P301S Transgenic Mice. PLoS ONE.

[70] Ho C.-Y., Troncoso J.C., Knox D., Stark W., Eberhart C.G. (2014). Beta-Amyloid, Phospho-Tau and Alpha-Synuclein Deposits Similar to Those in the Brain Are Not Identified in the Eyes of Alzheimer’s and Parkinson’s Disease Patients. Brain Pathol..

[71] López-Cuenca I., Marcos-Dolado A., Yus-Fuertes M., Salobrar-García E., Elvira-Hurtado L., Fernández-Albarral J.A., Salazar J.J., Ramírez A.I., Sánchez-Puebla L., Fuentes-Ferrer M.E. (2022). The Relationship Between Retinal Layers and Brain Areas in Asymptomatic First-Degree Relatives of Sporadic Forms of Alzheimer’s Disease: An Exploratory Analysis. Alzheimer’s Res. Ther..

[72] López-de-Eguileta A., López-García S., Lage C., Pozueta A., García-Martínez M., Kazimierczak M., Bravo M., Irure J., López-Hoyos M., Muñoz-Cacho P. (2022). The Retinal Ganglion Cell Layer Reflects Neurodegenerative Changes in Cognitively Unimpaired Individuals. Alzheimer’s Res. Ther..

[73] Eppenberger L.S., Li C., Wong D., Tan B., Garhöfer G., Hilal S., Chong E., Toh A.Q., Venketasubramanian N., Chen C.L.H. (2024). Retinal Thickness Predicts the Risk of Cognitive Decline over Five Years. Alzheimer’s Res. Ther..

[74] Lepoittevin M., Greig J., Erol O., Benani A., Bauvin P., Azzolini C., Donati S., Dubois B., Boscher C., Bodard S. (2026). Retinal Biomarkers for Early Alzheimer’s Detection: A Systematic Review of Optical Coherence Tomography (OCT) Findings. BMJ Open Ophthalmol..

[75] Jali V., Zhang Q., Chong J.R., Wong D., Tan B., Garhöfer G., Hilal S., Lai M.K.P., Schmetterer L., Chen C.L.-H. (2025). Diagnosis of Cognitive Impairment and Dementia: Blood Plasma and Optical Coherence Tomography. Brain Commun..

[76] Lad E.M., Mukherjee D., Stinnett S.S., Cousins S.W., Potter G.G., Burke J.R., Farsiu S., Whitson H.E. (2018). Evaluation of Inner Retinal Layers as Biomarkers in Mild Cognitive Impairment to Moderate Alzheimer’s Disease. PLoS ONE.

[77] Jáñez-Escalada L., Jáñez-García L., Salobrar-García E., Santos-Mayo A., de Hoz R., Yubero R., Gil P., Ramírez J.M. (2019). Spatial Analysis of Thickness Changes in Ten Retinal Layers of Alzheimer’s Disease Patients Based on Optical Coherence Tomography. Sci. Rep..

[78] Hood D.C., Raza A.S., de Moraes C.G.V., Liebmann J.M., Ritch R. (2013). Glaucomatous Damage of the Macula. Prog. Retin. Eye Res..

[79] Sen S., Saxena R., Tripathi M., Vibha D., Dhiman R. (2020). Neurodegeneration in Alzheimer’s Disease and Glaucoma: Overlaps and Missing Links. Eye.

[80] Nieves-Moreno M., Martínez-de-la-Casa J.M., Morales-Fernández L., Sánchez-Jean R., Sáenz-Francés F., García-Feijoó J. (2018). Impacts of Age and Sex on Retinal Layer Thicknesses Measured by Spectral Domain Optical Coherence Tomography with Spectralis. PLoS ONE.

[81] Romero-Bascones D., Ayala U., Alberdi A., Erramuzpe A., Galdós M., Gómez-Esteban J.C., Murueta-Goyena A., Teijeira S., Gabilondo I., Barrenechea M. (2022). Spatial Characterization of the Effect of Age and Sex on Macular Layer Thicknesses and Foveal Pit Morphology. PLoS ONE.

[82] Chua J., Li C., Antochi F., Toma E., Wong D., Tan B., Garhöfer G., Hilal S., Popa-Cherecheanu A., Chen C.L.-H. (2025). Utilizing Deep Learning to Predict Alzheimer’s Disease and Mild Cognitive Impairment with Optical Coherence Tomography. Alzheimer’s Dement..

[83] Turkan Y., Tek F.B., Arpaci F., Arslan O., Toslak D., Bulut M.T., Yaman A. (2024). Automated Diagnosis of Alzheimer’s Disease Using OCT and OCTA: A Systematic Review. IEEE Access.

[84] Wu H., Wang C., Chen C., Xu X., Zhu Y., Sang A., Jiang K., Dong J. (2020). Association Between Retinal Vascular Geometric Changes and Cognitive Impairment: A Systematic Review and Meta-Analysis. J. Clin. Neurol..

[85] Mathew S., Huang Y.N., Bice P., Saykin A.J., Risacher S.L. (2025). Retinal Vascular Biomarkers in Mild Cognitive Impairment and Alzheimer’s Disease: A Comprehensive Review and Meta-Analysis. Alzheimer’s Dement..

[86] Chua J., Singh S., Gong S., Wong D., Tan B., Toh A.Q., Venketasubramanian N., Tan B.Y., Chong J.R., Lai M.K.P. (2026). Combining OCT and OCT Angiography Improves Differentiation of Mild Cognitive Impairment and Alzheimer’s Disease. Alzheimer’s Res. Ther..

[87] Querques G., Borrelli E., Sacconi R., De Vitis L., Leocani L., Santangelo R., Magnani G., Comi G., Bandello F. (2019). Functional and Morphological Changes of the Retinal Vessels in Alzheimer’s Disease and Mild Cognitive Impairment. Sci. Rep..

[88] Lee S.J.V., Goh Y.Q., Rojas-Carabali W., Cifuentes-González C., Cheung C.Y., Arora A., de-la-Torre A., Gupta V., Agrawal R. (2025). Association Between Retinal Vessels Caliber and Systemic Health: A Comprehensive Review. Surv. Ophthalmol..

[89] Jack C.R., Bennett D.A., Blennow K., Carrillo M.C., Dunn B., Budd Haeberlein S., Holtzman D.M., Jagust W.J., Jessen F., Karlawish J. (2018). NIA-AA Research Framework: Toward a Biological Definition of Alzheimer’s Disease. Alzheimer’s Dement..

[90] Chun M.Y., Jang H., Kim S.-J., Park Y.H., Yun J., Lockhart S.N., Weiner M., De Carli C., Moon S.H., Choi J.Y. (2024). Emerging Role of Vascular Burden in AT(N) Classification in Individuals with Alzheimer’s and Concomitant Cerebrovascular Burdens. J. Neurol. Neurosurg. Psychiatry.

[91] Iturria-Medina Y., Sotero R.C., Toussaint P.J., Mateos-Pérez J.M., Evans A.C. (2016). Early Role of Vascular Dysregulation on Late-Onset Alzheimer’s Disease Based on Multifactorial Data-Driven Analysis. Nat. Commun..

