# Supplementary material for: Multimodal Retinal Imaging for the Detection of Early Alzheimer’s Disease: Combining Biochemical, Structural, and Vascular Biomarkers
Source: Bioengineering (Basel). 2026 Sep 8;13(9):1041. doi: 10.3390/bioengineering13091041 (PMC13603378; doi:10.3390/bioengineering13091041)
Supplement: Supplementary file 1 [file bioengineering-13-01041-s001.zip › File S1 Electronic supplementary material.pdf]

Electronic supplementary material

Supplementary Methods

Fold-Internal HSI Wavelength-Selection Sensitivity Analysis: The HSI predictor used in the primary multimodal analyses was identified during an exploratory full-cohort biomarker screen. To quantify the potential optimism introduced by this data-dependent wavelength selection, an additional fold-internal sensitivity analysis was performed.

Within each outer LOOCV fold, wavelength selection was repeated using only the training participants, with the held-out participant excluded from the selection procedure. The wavelength showing the strongest training-set discrimination was then used to generate the prediction for the held-out participant. This procedure was performed separately for the full macular grid, inferior hemisphere, and superior hemisphere.

GC-IPL Dimensionality-Reduction Sensitivity Analyses: Because the primary GC-IPL model included thickness measurements from all nine ETDRS regions, three alternative modelling strategies were evaluated to assess the effect of predictor dimensionality.

First, the nine regional GC-IPL measurements were modelled using elastic-net logistic regression. The regularization parameters were selected using an inner cross-validation procedure restricted to the training data of each outer LOOCV fold.

Second, principal component analysis (PCA) was performed within each outer training fold, with the number of components selected to retain approximately 90% of the variance. The fitted transformation was subsequently applied to the held-out participant before logistic regression classification.

Third, the nine regional measurements were collapsed into a single global-mean GC-IPL feature, computed as the row-wise mean across all nine regions, and this single feature replaced the regional values in an otherwise identical logistic-regression classifier. As this transformation involves no fitted parameters, no fold-internal restriction was required.

Performance of all three sensitivity models was compared with that of the original nine-region GC-IPL model using paired subject-level bootstrap differences in AUC.

Covariate-Augmented Sensitivity Analyses: To evaluate whether demographic or genetic factors materially influenced classification performance, the principal HSI-only, GC-IPL-only, and HSI+GC-IPL models were refitted after adding age, sex, and APOE ε4 carrier status as covariates.

These covariates were included within the same LOOCV framework as the primary models, with all preprocessing performed using training data only. Changes in AUC relative to the corresponding models without covariates were evaluated using paired subject-level bootstrap analysis.

Supplementary Results

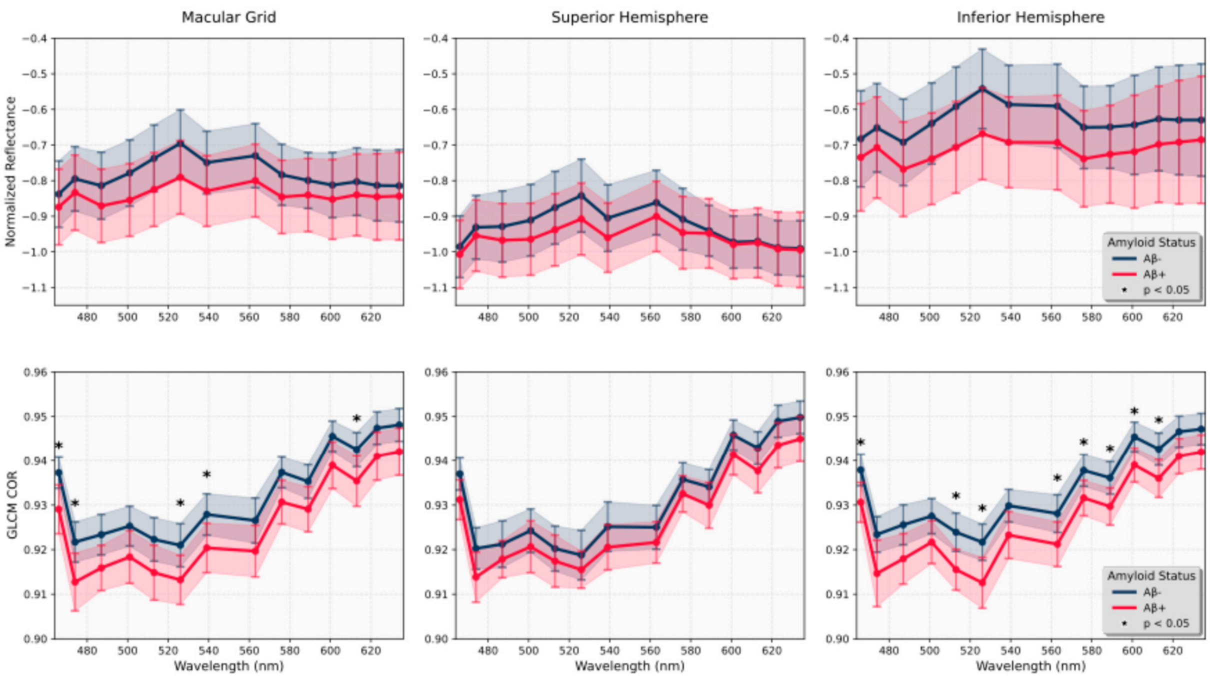

Supplementary Figure S1: Spectral reflectance and texture features across macular grids by amyloid status. Top row: Mean normalized reflectance spectra with 95% confidence intervals for Aβ− (blue) and Aβ+ (red) participants, shown for the full macular circle (left), superior hemisphere (middle), and inferior hemisphere (right). Reflectance curves show substantial overlap between groups across all regions and wavelengths. Bottom row: Corresponding GLCM COR values at each wavelength. Significant group differences ($p < 0.05$; black asterisks, Mann-Whitney U test) were observed at multiple wavelengths in the full macular grid and inferior hemisphere regions, with the most pronounced difference at 466 nm ($p = 0.03$ and $p = 0.01$, respectively). *Aβ = amyloid-beta; GLCM = gray-level co-occurrence matrix; COR = correlation.*

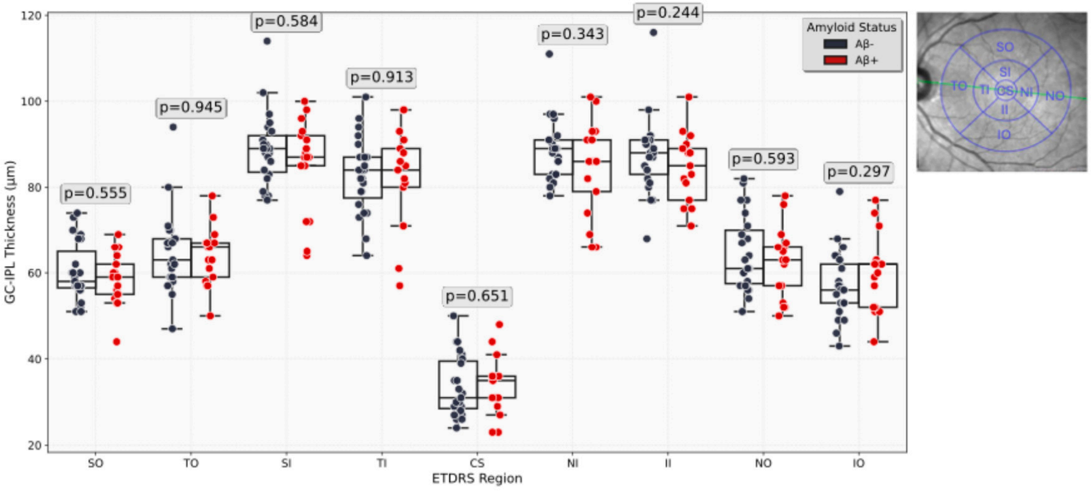

Supplementary Figure S2: GC-IPL thickness by ETDRS region and amyloid status Box plots show the distribution of GC-IPL thickness across nine ETDRS regions for Aβ− (blue) and Aβ+ (red) participants. *P* values are computed using the Mann-Whitney U test. *GC-IPL = ganglion cell–inner plexiform layer; ETDRS = Early Treatment Diabetic Retinopathy Study; Aβ = amyloid-beta*.

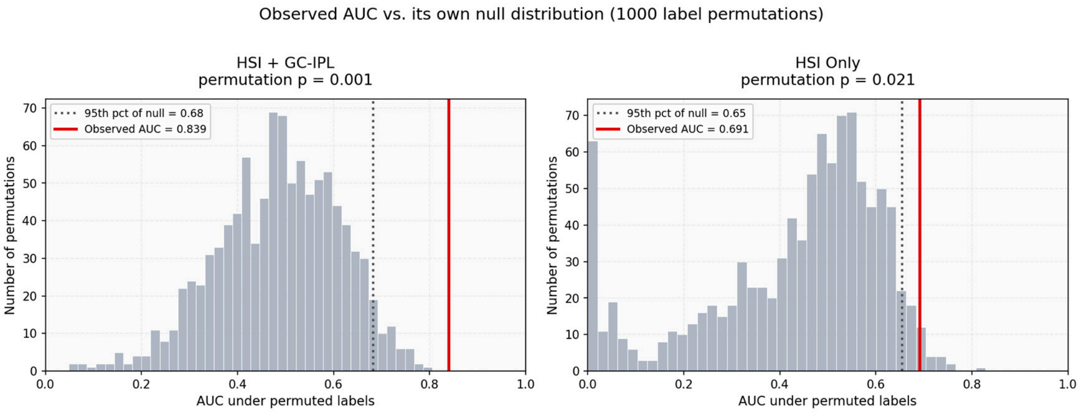

Supplementary Figure S3: Permutation test, visualized Observed AUC plotted against its model-specific null distribution (1,000 label permutations) for each of the seven primary classification models. Grey values represent null AUCs, the dotted line denotes the 95th percentile of the corresponding null distribution, and the red line indicates the observed AUC. *AUC = area under the receiver operating characteristic curve.*

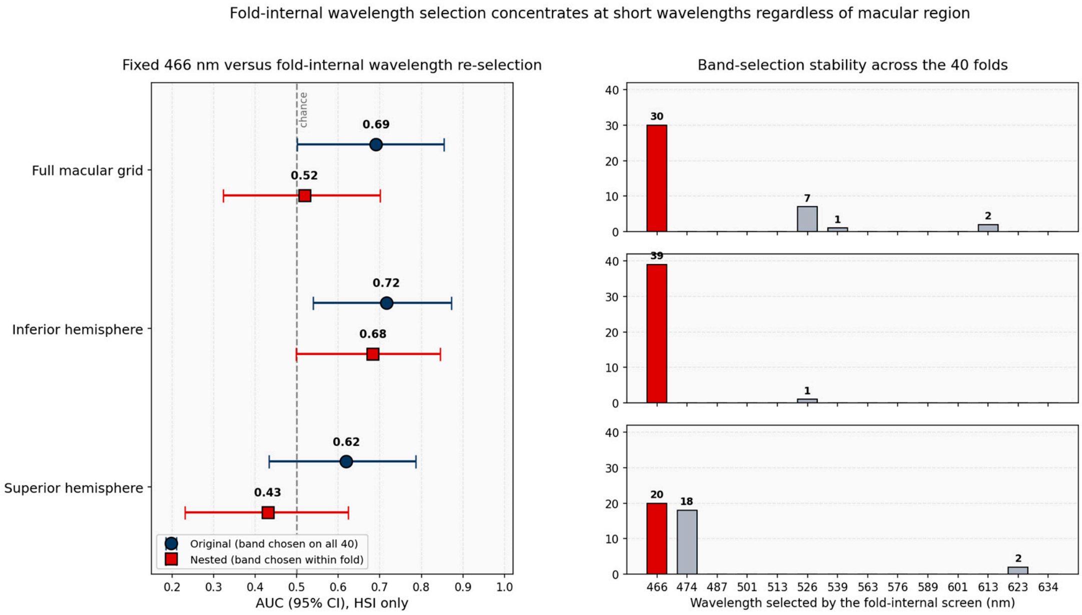

Supplementary Figure S4: Sensitivity of HSI classification performance to the wavelength-selection procedure, and stability of fold-internal wavelength selection, by macular region
Left: fixed 466-nm candidate feature versus fold-internal wavelength re-selection, HSI-only AUC with 95% CIs, shown separately for the full macular grid, inferior hemisphere, and superior hemisphere. Right: the wavelength selected by the fold-internal screen in each of the 40 outer folds, by region. 466 nm was selected in 30/40 folds (75%) for the full macular grid and 39/40 folds (97.5%) for the inferior hemisphere. *HSI = hyperspectral imaging; AUC = area under the curve; CI = confidence interval.*

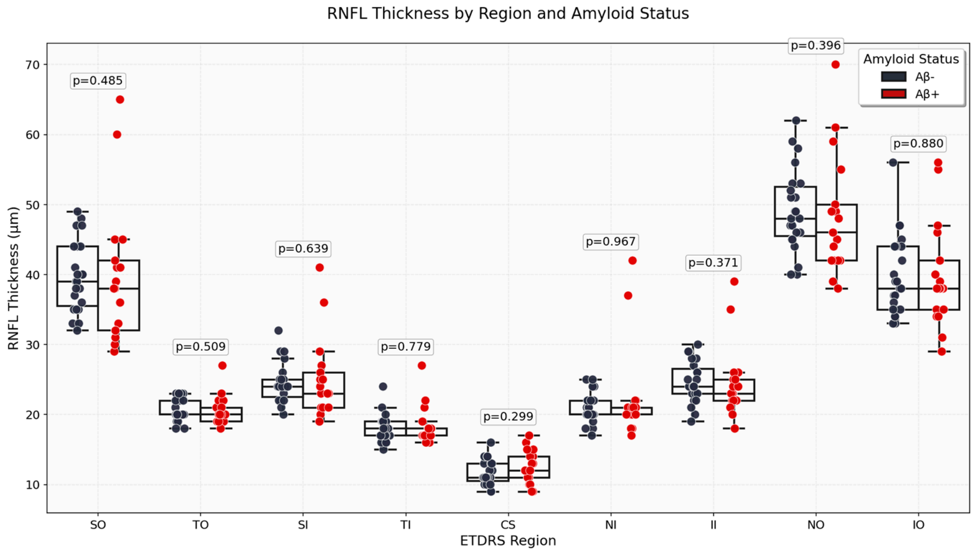

Supplementary Figure S5: RNFL thickness by ETDRS region and amyloid status Box plots show the distribution of RNFL thickness across nine ETDRS regions for Aβ− (blue) and Aβ+ (red) participants, using the same region ordering and presentation as Supplementary Figure 2. Regional distributions overlapped substantially between groups; descriptive statistics are reported in Supplementary Table 9 without additional inferential testing. *RNFL = retinal nerve fiber layer; ETDRS = Early Treatment Diabetic Retinopathy Study; Aβ = amyloid-beta.*

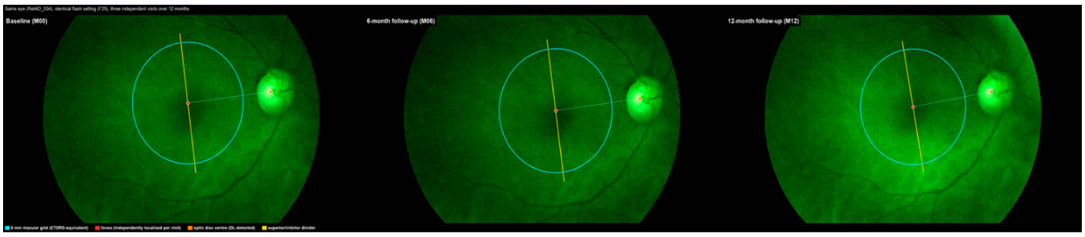

Supplementary Figure S6: Repeat-visit macular grid alignment HSI reconstructions from the same eye at baseline, 6 months, and 12 months, acquired using the same flash setting. The fovea and optic-disc landmarks were localized independently at each visit, and the corresponding 6-mm macular grid was reconstructed separately for each acquisition. The close alignment of the grid relative to the retinal vasculature illustrates the practical consistency of repeated landmark placement across visits.

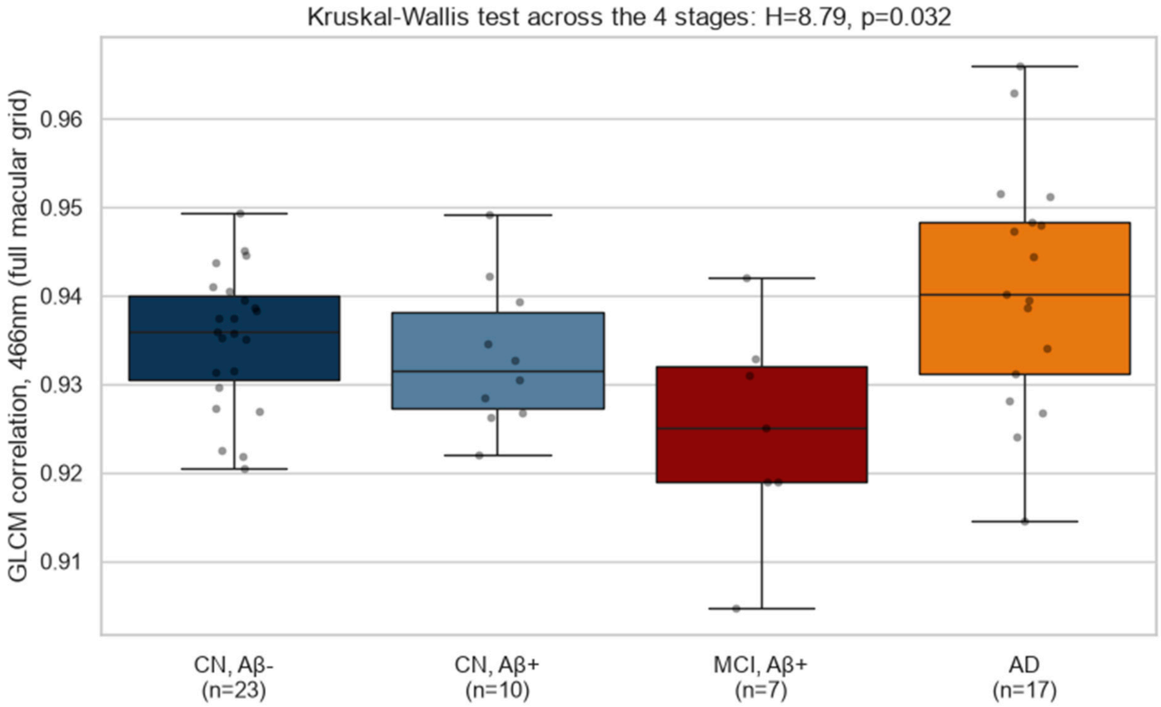

Supplementary Figure S7: GLCM correlation (466 nm, full macular grid) across four disease-stage groups spanning both cohorts: CN,Aβ− (n = 23), CN,Aβ+ (n = 10), and MCI,Aβ+ (n = 7), ADD (n = 17). Box plots show the median and interquartile range; points show individual participants. Kruskal–Wallis test across the four groups: H = 8.79, p = 0.032.

Supplementary Table S1: Demographic characteristics of the combined cohorts

**Group**	**CN**	**CN/MCI (Aβ+)**	**ADD**	***P* values**
**Total Participants**	45	17	17	-
**Sex (M/F)**	19/26	5/12	7/10	0.428‡
**Age (Mean ± SD)**	72.6 ± 8.6	73.8 ± 7.6	71.9 ± 6.6	0.644*

*Aβ+ = amyloid-beta positive; SD = standard deviation; M = male; F = female; CN = cognitively normal; MCI = mild cognitive impairment; ADD = Alzheimer's disease dementia; ‡Chi-square test ; *Kruskal-Wallis test.*

Supplementary Table S2: Summary of the VBMs between amyloid groups extracted from CFP and computed using the PVBM toolbox Biomarkers are grouped by vessel type (artery and vein). For each biomarker, the mean and SD are presented, along with p-values calculated using two-sided Mann-Whitney U tests to assess group differences. Nominal p-values < 0.05 are highlighted in bold. Benjamini–Hochberg FDR-adjusted q-values are also reported.

**Vascular Biomarker**	**Aβ- (n = 23)**		**Aβ+ (n = 17)**		**MWU test**	**BH FDR**
	**Mean**	**SD**	**Mean**	**SD**	***P* values**	***q-value***
Artery						
Area	15509.783	2396.691	15758.471	1730.071	0.702	0.956
TI	1.079	0.017	1.081	0.023	0.956	0.956
TOR	1.075	0.009	1.075	0.006	0.784	0.956
OVLEN	1352.321	245.434	1333.621	211.190	0.935	0.956
START	7.739	1.322	7.798	1.425	0.856	0.956
END	9.087	1.443	9.726	1.564	0.247	0.956
INTER	1.739	1.214	1.882	0.928	0.658	0.956
D_0	1.014	0.038	1.015	0.032	0.805	0.956
D_1	0.975	0.046	0.973	0.037	0.913	0.956
D_2	0.959	0.047	0.956	0.038	0.891	0.956
SL	1.159	0.361	1.237	0.298	0.603	0.956

CRAE (Knudtson)	21.957	2.569	23.988	2.527	**0.031**	0.519
Vein						
Area	16766.000	2350.104	17554.398	2173.426	0.147	0.956
TI	1.075	0.013	1.071	0.009	0.494	0.956
medTor	1.073	0.009	1.071	0.010	0.529	0.956
Ovlen	1222.221	214.711	1262.038	233.345	0.511	0.956
Startp	7.043	1.147	7.724	1.440	0.208	0.956
Endp	8.087	0.848	9.038	1.523	**0.043**	0.519
Interp	1.043	1.107	1.251	0.750	0.198	0.956
D_0	1.007	0.027	1.006	0.035	0.870	0.956
D_1	0.959	0.028	0.961	0.042	0.827	0.956
D_2	0.939	0.029	0.942	0.042	0.722	0.956
SL	1.245	0.299	1.271	0.304	0.784	0.956
CRVE (Knudtson)	35.008	4.776	35.832	2.998	0.662	0.956

VBM = vascular biomarker; CFP = color fundus photography; *PVBM = Python Vasculature BioMarker; Aβ+ = amyloid-beta positive; Aβ- = amyloid-beta negative; SD = standard deviation; MWU = Mann–Whitney U test; TI = tortuosity index; TOR = median tortuosity; OVLEN = overall length; START = number of starting points; END = number of end points; INTER = number of intersection points; D0 = capacity dimension; D1 = entropy dimension; D2 = correlation dimension; SL = singularity length; CRAE = central retinal arteriolar equivalent, CRVE = central retinal venular equivalent*

Supplementary Table S3: Classification performance metrics for different feature set combinations across macular regions. For each combination, the number of features, AUC with 95% confidence intervals, accuracy, sensitivity, specificity, and F1 score are reported. The best results per region are highlighted in **bold**. *N Features = number of features; AUC = Area Under Curve; CI = confidence interval; HSI = hyperspectral imaging; VBMs = vascular biomarkers; GC-IPL = ganglion cell–inner plexiform layer.*

Full Macular grid							
**Feature Set**	**N Features**	**AUC**	**AUC 95% CI**	**Accuracy**	**Sensitivity**	**Specificity**	**F1-score**
HSI Only	1	0.691	0.501-0.854	0.700	0.706	0.696	0.667
VBMs Only	2	0.675	0.499-0.843	0.650	0.647	0.652	0.611
GC-IPL Only	9	0.731	0.562-0.875	0.600	0.529	0.652	0.529
HSI + VBMs	3	0.703	0.521-0.848	0.625	0.588	0.652	0.571
**HSI + GC-IPL**	10	**0.839**	**0.670-0.969**	**0.800**	**0.824**	**0.783**	**0.778**
VBMs + GC-IPL	11	0.749	0.570-0.886	0.700	0.706	0.696	0.667
HSI + VBMs + GC-IPL	12	0.808	0.624-0.951	**0.800**	**0.824**	**0.783**	**0.778**
**Superior Hemisphere**							
**Feature Set**	**N Features**	**AUC**	**AUC 95% CI**	**Accuracy**	**Sensitivity**	**Specificity**	**F1- score**
HSI Only	1	0.619	0.433-0.787	0.550	0.471	0.609	0.471
VBMs Only	2	0.675	0.499-0.843	0.650	0.647	0.652	0.611
GC-IPL Only	2	0.448	0.246-0.645	0.425	0.412	0.435	0.378
HSI + VBMs	3	0.683	0.505-0.846	0.650	**0.706**	0.609	**0.632**
HSI + GC-IPL	3	0.598	0.406-0.771	0.500	0.471	0.522	0.444
VBMs + GC-IPL	4	0.680	0.494-0.845	0.575	0.588	0.565	0.541
HSI + VBMs + GC-IPL	5	**0.721**	**0.538-0.870**	**0.675**	0.647	**0.696**	0.629
**Inferior Hemisphere**							
**Feature Set**	**N Features**	**AUC**	**AUC 95% CI**	**Accuracy**	**Sensitivity**	**Specificity**	**F1-score**
HSI Only	1	0.716	0.539-0.872	**0.700**	0.647	**0.739**	**0.647**

VBMs Only	2	0.675	0.499-0.843	0.650	0.647	0.652	0.611
GC-IPL Only	2	0.708	0.539-0.865	0.575	0.529	0.609	0.514
HSI + VBMs	3	0.683	0.502-0.833	0.600	0.529	0.652	0.529
HSI + GC-IPL	3	0.754	0.593-0.897	0.650	0.647	0.652	0.611
VBMs + GC-IPL	4	0.760	0.594-0.908	0.675	**0.706**	0.652	0.649
HSI + VBMs + GC-IPL	5	**0.788**	**0.621-0.931**	0.625	0.647	0.609	0.595

Supplementary Table S4: Classification performance, statistical support, and significance for all seven reported models, ranked by AUC. Predictors fitted is the number of parameters the model estimates. EPV (events per predictor parameter) is the number of Aβ+ cases (17) divided by predictors fitted. AUC (95% CI) is estimated by leave-one-subject-out cross-validation, with the 95% CI from 1,000 bootstrap resamples. Permutation p is the empirical p-value from 1,000 label permutations, each re-running the full LOOCV pipeline on shuffled Aβ labels. ΔAUC vs. HSI+GC-IPL (95% CI) and its accompanying p give the paired-bootstrap difference in AUC between each model and the HSI+GC-IPL model, computed on the same 1,000 subject-level resamples; the HSI+GC-IPL row is the reference and carries no self-comparison. *HSI = hyperspectral imaging; GC-IPL = ganglion cell–inner plexiform layer; VBMs = vascular biomarkers; EPV = events per predictor parameter; AUC = area under the receiver operating characteristic curve; CI = confidence interval.*

**Model**	**Predictors fitted**	**EPV**	**AUC (95% CI)**	**Permutation p**	**ΔAUC vs. HSI+GC-IPL (95% CI)**	**p**
HSI + GC-IPL	10	1.70	0.839 (0.670–0.969)	0.001	—	—

HSI + VBMs + GC-IPL	12	1.42	0.808 (0.624–0.951)	0.003	−0.031 (−0.094 to 0.027)	0.310
VBMs + GC-IPL	11	1.55	0.749 (0.570–0.886)	0.012	−0.090 (−0.224 to 0.073)	0.250
GC-IPL only	9	1.89	0.731 (0.562–0.875)	0.019	−0.107 (−0.253 to 0.039)	0.172
HSI + VBMs	3	5.67	0.703 (0.521–0.848)	0.027	−0.136 (−0.310 to 0.038)	0.102
HSI only	1	17.00	0.691 (0.501–0.854)	0.021	−0.148 (−0.302 to 0.009)	0.064
VBMs only	2	8.50	0.675 (0.499–0.843)	0.040	−0.164 (−0.362 to 0.070)	0.148

Supplementary Table S5: Fold-internal wavelength-selection stability by macular region

Wavelength selected by the fold-internal screen (Supplementary Methods) in each of the 40 outer LOOCV folds, by macular region. *LOOCV = leave-one-subject-out cross-validation.*

**Region**	**Selected band**	**Folds selected**	**Fraction of folds**
Full macular grid	466 nm	30	0.750
Full macular grid	526 nm	7	0.175
Full macular grid	613 nm	2	0.050
Full macular grid	539 nm	1	0.025
Inferior hemisphere	466 nm	39	0.975

Inferior hemisphere	526 nm	1	0.025
Superior hemisphere	466 nm	20	0.500
Superior hemisphere	474 nm	18	0.450
Superior hemisphere	623 nm	2	0.050

Supplementary Table S6: GC-IPL dimensionality-reduction sensitivity models. The original nine-region GC-IPL model was refit using elastic-net regularization; separately, using fold-internal PCA (retaining ~90% of variance), with all tuning performed inside the outer training fold; and separately, by collapsing the nine regions into a single global-mean feature. ΔAUC and p are paired-bootstrap comparisons against the original nine-region GC-IPL model. GC-IPL = ganglion cell–inner plexiform layer; PCA = principal component analysis; EPV = events per predictor parameter; AUC = area under the curve; CI = confidence interval.

**Model**	**N features (fitted)**	**EPV**	**AUC (95% CI)**	**Sensitivity**	**Specificity**	**ΔAUC vs original (95% CI)**	**p**
GC-IPL only (original, 9 regions)	9	1.89	0.731 (0.562–0.875)	0.529	0.652	—	—
GC-IPL only (elastic-net)	9	1.89	0.726 (0.552–0.875)	0.471	0.652	−0.005 (−0.071 to 0.057)	0.944

GC-IPL only (fold-internal PCA, ~90%)	4	4.25	0.606 (0.429–0.783)	0.588	0.609	−0.125 (−0.251 to 0.025)	0.108
GC-IPL only (global mean of 9 regions)	1	17.00	0.450 (0.260–0.637)	0.529	0.522	−0.281 (−0.501 to −0.058)	0.012

Supplementary Table S7: Covariate-augmented sensitivity models (age, sex, APOE ε4) Each principal model was refit after adding age, sex, and APOE ε4 carrier status as covariates within the same LOOCV framework. ΔAUC and p are paired-bootstrap comparisons against the corresponding model without covariates. *APOE = apolipoprotein E; EPV = events per predictor parameter; AUC = area under the curve; CI = confidence interval; LOOCV = leave-one-subject-out cross-validation.*

**Model**	**N features (fitted)**	**EPV**	**AUC (95% CI)**	**ΔAUC vs base model (95% CI)**	**p**
HSI only + covariates	4	4.25	0.624 (0.417–0.807)	−0.066 (−0.170 to 0.028)	0.204
GC-IPL only + covariates	12	1.42	0.754 (0.593–0.901)	+0.023 (−0.128 to 0.193)	0.730
HSI + GC-IPL + covariates	13	1.31	0.772 (0.593–0.920)	−0.066 (−0.193 to 0.057)	0.358

Supplementary Table S8: Benjamini–Hochberg FDR-adjusted q-values by feature family. Raw p<0.05 is the number of comparisons within the family with nominal p<0.05; BH q<0.05 is the number remaining significant after Benjamini–Hochberg correction within that family; smallest q is the minimum FDR-adjusted q-value observed in the family. Individual nominal p-values and BH-adjusted q-values for every comparison within each family are given in Supplementary Table 2 (vascular biomarkers), Supplementary Table 9 (RNFL), and Supplementary Tables 10–12 (HSI GLCM correlation, HSI normalized reflectance, and GC-IPL, respectively). *FDR = false discovery rate; GLCM = gray-level co-occurrence matrix; COR = correlation; GC-IPL = ganglion cell–inner plexiform layer; RNFL = retinal nerve fiber layer.*

**Feature family**	**Tests**	**Raw p<0.05**	**BH q<0.05**	**Smallest q**
HSI GLCM COR screen (14 bands × 3 regions)	42	13	0	0.140
HSI normalized reflectance (14 bands × 3 regions)	42	0	0	0.913
Vascular biomarkers (24 parameters)	24	2	0	0.519
GC-IPL (9 ETDRS regions)	9	0	0	0.837
RNFL (9 ETDRS regions)	9	0	0	0.916

Supplementary Table S9: RNFL descriptive statistics by ETDRS region Mean ± SD RNFL thickness (µm) by ETDRS region and amyloid status, with nominal p-values (Mann–Whitney U test) and Benjamini–Hochberg FDR-adjusted q-values. *RNFL = retinal nerve fiber layer; ETDRS = Early Treatment Diabetic Retinopathy Study; SD = standard deviation; BH = Benjamini–Hochberg; Aβ = amyloid-beta*.

**Region**	**Aβ− (n = 23)**	**Aβ+ (n = 17)**	**p**	**BH q**
Central subfield	11.74 ± 1.84	12.53 ± 2.40	0.299	0.916
Inner inferior	24.61 ± 2.94	24.59 ± 5.16	0.371	0.916
Outer inferior	39.65 ± 5.71	39.71 ± 7.54	0.880	0.967
Inner temporal	17.91 ± 1.95	18.41 ± 2.72	0.779	0.967
Outer temporal	20.78 ± 1.62	20.65 ± 2.09	0.509	0.916
Inner superior	24.52 ± 2.81	25.12 ± 5.72	0.639	0.959
Outer superior	39.74 ± 5.06	39.82 ± 9.95	0.485	0.916
Inner nasal	20.91 ± 2.13	22.47 ± 6.62	0.967	0.967
Outer nasal	49.13 ± 5.87	48.29 ± 8.59	0.396	0.916

Supplementary Table S10: HSI GLCM correlation — nominal p-values and Benjamini–Hochberg-adjusted q-values by wavelength and macular region Median values and nominal p-values (Mann–Whitney U test) reproduce the per-wavelength comparisons underlying Supplementary Figure 1 (bottom row); q-values are Benjamini–Hochberg FDR-adjusted within this 42-comparison family (see Supplementary Table 8). *HSI = hyperspectral imaging; GLCM = gray-level co-occurrence matrix; COR = correlation; FDR = false discovery rate; Aβ = amyloid-beta*.

**Region**	**Wavelength (nm)**	**Median Aβ−**	**Median Aβ+**	**p**	**q**
Full macular grid	466	0.939	0.931	0.031	0.140
Full macular grid	474	0.922	0.914	0.046	0.140
Full macular grid	487	0.925	0.918	0.063	0.140
Full macular grid	501	0.926	0.923	0.113	0.163
Full macular grid	513	0.924	0.916	0.132	0.185
Full macular grid	526	0.921	0.916	0.038	0.140
Full macular grid	539	0.928	0.920	0.049	0.140
Full macular grid	563	0.928	0.920	0.106	0.160
Full macular grid	576	0.938	0.930	0.067	0.140
Full macular grid	589	0.936	0.928	0.085	0.148
Full macular grid	601	0.947	0.938	0.071	0.142
Full macular grid	613	0.944	0.937	0.043	0.140
Full macular grid	623	0.950	0.940	0.080	0.148
Full macular grid	634	0.950	0.941	0.090	0.151

Superior hemisphere	466	0.939	0.935	0.106	0.160
Superior hemisphere	474	0.919	0.910	0.106	0.160
Superior hemisphere	487	0.919	0.921	0.262	0.297
Superior hemisphere	501	0.925	0.922	0.444	0.454
Superior hemisphere	513	0.923	0.919	0.511	0.511
Superior hemisphere	526	0.918	0.913	0.171	0.218
Superior hemisphere	539	0.926	0.921	0.218	0.262
Superior hemisphere	563	0.925	0.919	0.274	0.303
Superior hemisphere	576	0.935	0.933	0.367	0.385
Superior hemisphere	589	0.934	0.932	0.298	0.321
Superior hemisphere	601	0.945	0.943	0.171	0.218

Superior hemisphere	613	0.943	0.941	0.218	0.262
Superior hemisphere	623	0.947	0.945	0.147	0.199
Superior hemisphere	634	0.947	0.946	0.229	0.267
Inferior hemisphere	466	0.940	0.932	0.010	0.140
Inferior hemisphere	474	0.924	0.921	0.067	0.140
Inferior hemisphere	487	0.926	0.919	0.059	0.140
Inferior hemisphere	501	0.929	0.925	0.085	0.148
Inferior hemisphere	513	0.925	0.915	0.023	0.140
Inferior hemisphere	526	0.921	0.914	0.023	0.140
Inferior hemisphere	539	0.932	0.925	0.055	0.140
Inferior hemisphere	563	0.930	0.924	0.049	0.140

Inferior hemisphere	576	0.940	0.933	0.033	0.140
Inferior hemisphere	589	0.938	0.931	0.043	0.140
Inferior hemisphere	601	0.946	0.939	0.017	0.140
Inferior hemisphere	613	0.944	0.935	0.025	0.140
Inferior hemisphere	623	0.948	0.940	0.052	0.140
Inferior hemisphere	634	0.949	0.941	0.055	0.140

Supplementary Table S11: HSI normalized reflectance — nominal p-values and Benjamini–Hochberg-adjusted q-values by wavelength and macular region Median values and nominal p-values (Mann–Whitney U test) reproduce the per-wavelength comparisons underlying Supplementary Figure 1 (top row); q-values are Benjamini–Hochberg FDR-adjusted within this 42-comparison family (see Supplementary Table 8). *HSI = hyperspectral imaging; FDR = false discovery rate; Aβ = amyloid-beta.*

**Region**	**Wavelength (nm)**	**Median Aβ−**	**Median Aβ+**	**p**	**q**
Full macular grid	466	-0.859	-0.907	0.848	0.913
Full macular grid	474	-0.828	-0.840	0.763	0.913

Full macular grid	487	-0.875	-0.891	0.566	0.913
Full macular grid	501	-0.835	-0.893	0.412	0.913
Full macular grid	513	-0.786	-0.878	0.311	0.913
Full macular grid	526	-0.757	-0.844	0.208	0.913
Full macular grid	539	-0.817	-0.863	0.367	0.913
Full macular grid	563	-0.773	-0.811	0.477	0.913
Full macular grid	576	-0.790	-0.837	0.511	0.913
Full macular grid	589	-0.783	-0.861	0.642	0.913
Full macular grid	601	-0.837	-0.823	0.763	0.913
Full macular grid	613	-0.803	-0.807	0.827	0.913
Full macular grid	623	-0.837	-0.832	0.913	0.935
Full macular grid	634	-0.838	-0.845	0.848	0.913
Superior hemisphere	466	-1.016	-1.092	0.784	0.913
Superior hemisphere	474	-0.980	-1.012	0.603	0.913
Superior hemisphere	487	-0.992	-1.024	0.642	0.913
Superior hemisphere	501	-0.964	-1.020	0.460	0.913
Superior hemisphere	513	-0.913	-0.999	0.396	0.913

Superior hemisphere	526	-0.874	-0.991	0.412	0.913
Superior hemisphere	539	-0.994	-1.055	0.338	0.913
Superior hemisphere	563	-0.914	-0.995	0.477	0.913
Superior hemisphere	576	-0.946	-1.039	0.566	0.913
Superior hemisphere	589	-0.976	-1.017	0.805	0.913
Superior hemisphere	601	-0.998	-0.949	0.848	0.913
Superior hemisphere	613	-0.986	-0.976	0.870	0.913
Superior hemisphere	623	-0.984	-1.003	0.870	0.913
Superior hemisphere	634	-1.001	-0.999	0.870	0.913
Inferior hemisphere	466	-0.645	-0.712	0.763	0.913
Inferior hemisphere	474	-0.602	-0.684	0.827	0.913

Inferior hemisphere	487	-0.662	-0.813	0.702	0.913
Inferior hemisphere	501	-0.638	-0.767	0.381	0.913
Inferior hemisphere	513	-0.570	-0.725	0.298	0.913
Inferior hemisphere	526	-0.534	-0.676	0.262	0.913
Inferior hemisphere	539	-0.586	-0.711	0.367	0.913
Inferior hemisphere	563	-0.563	-0.648	0.412	0.913
Inferior hemisphere	576	-0.664	-0.761	0.529	0.913
Inferior hemisphere	589	-0.740	-0.696	0.682	0.913
Inferior hemisphere	601	-0.773	-0.712	0.870	0.913
Inferior hemisphere	613	-0.732	-0.693	0.935	0.935
Inferior hemisphere	623	-0.699	-0.686	0.870	0.913

Inferior hemisphere	634	-0.677	-0.679	0.848	0.913

Supplementary Table S12: GC-IPL descriptive statistics by ETDRS region Mean ± SD GC-IPL thickness (µm) by ETDRS region and amyloid status, with nominal p-values (Mann–Whitney U test) and Benjamini–Hochberg FDR-adjusted q-values, reproducing the per-region comparisons underlying Supplementary Figure 2 (see Supplementary Table 8). *GC-IPL = ganglion cell–inner plexiform layer; ETDRS = Early Treatment Diabetic Retinopathy Study; SD = standard deviation; BH = Benjamini–Hochberg; Aβ = amyloid-beta*.

**Region**	**Aβ− (n = 23)**	**Aβ+ (n = 17)**	**p**	**BH q**
Central subfield	33.56 ± 7.08	33.71 ± 6.68	0.651	0.837
Inner inferior	87.56 ± 8.97	84.29 ± 7.97	0.244	0.837
Outer inferior	57.13 ± 7.90	60.06 ± 8.63	0.297	0.837
Inner temporal	82.74 ± 8.87	82.12 ± 11.57	0.913	0.945
Outer temporal	64.65 ± 9.36	64.00 ± 6.67	0.945	0.945
Inner superior	88.83 ± 8.40	85.35 ± 10.83	0.584	0.837
Outer superior	60.22 ± 6.86	58.35 ± 6.09	0.555	0.837
Inner nasal	88.61 ± 7.14	84.41 ± 10.68	0.343	0.837
Outer nasal	64.56 ± 8.88	62.29 ± 8.08	0.593	0.837
